# Soil pH modulates microbial nitrogen allocation in soil via compositional and metabolic shifts across forests in Japan

**DOI:** 10.1002/imo2.70054

**Published:** 2025-09-18

**Authors:** Yaping Liu, Yuta Ise, Hideto Takami, Rieko Urakawa, Ryunosuke Tateno, Atsushi Toyoda, Nobuhito Ohte, Weiyu Shi, Lin Jiang, Kazuo Isobe

**Affiliations:** ^1^ Institute of Ecology, College of Urban and Environmental Sciences Peking University Beijing China; ^2^ State Key Laboratory of Vegetation Structure, Function and Construction (VegLab) Peking University Beijing China; ^3^ Graduate School of Agricultural and Life Sciences The University of Tokyo Tokyo Japan; ^4^ Institute for Agro‐Environmental Sciences National Agriculture and Food Research Organization Ibaraki Japan; ^5^ Faculty of Agriculture Tokyo University of Agriculture and Technology Tokyo Japan; ^6^ Center for Mathematical Science and Advanced Technology, JAMSTEC Yokohama Japan; ^7^ Asia Center for Air Pollution Research Japan Environmental Sanitation Center Niigata Japan; ^8^ Field Science Education and Research Center Kyoto University Kyoto Japan; ^9^ Comparative Genomics Laboratory National Institute of Genetics Shizuoka Japan; ^10^ Graduate School of Informatics Kyoto University Kyoto Japan; ^11^ School of Geographical Sciences Southwest University Chongqing China; ^12^ School of Biological Sciences Georgia Institute of Technology Atlanta Georgia USA

**Keywords:** ammonification, forest soil, metagenome, N retention, N release

## Abstract

Ammonium release (ammonification) and uptake (immobilization) by soil microbial communities are fundamental processes of forest nitrogen (N) cycling, representing major N fluxes that influence plant productivity and ecosystem N retention. However, because these processes involve diverse metabolic pathways distributed across many taxa, they are difficult to evaluate using gene‐ or taxon‐specific approaches, and it remains unclear how microbial community structure governs the patterns of these processes. In this study, we examined how the abundance, taxonomic composition, richness, and metabolic capabilities of microbial communities regulate ammonium‐related N cycling processes across a wide range of forests in Japan, using rRNA gene sequencing and quantification, shotgun metagenomics, and ^¹⁵^N tracer assays. Across the full gradients of soil pH and N content, microbial abundance was primarily correlated with the absolute rates of N cycling processes, while taxonomic composition and richness were more strongly correlated with N allocation—that is, the balance among ammonium release, ammonium uptake, and subsequent nitrification. Soils with higher pH supported taxonomic compositions linked to enhanced ammonium release and nitrification, whereas lower‐pH soils hosted compositions associated with greater ammonium uptake and retention. Notably, the regulatory influence of taxonomic composition on N allocation was pronounced within the higher‐pH range but diminished within the lower‐pH range. Despite this environmental dependency, N allocation by soil microbial communities was ultimately constrained by their overall metabolic capabilities. In higher‐pH soils, microbial communities were enriched in metabolic functions related to nutrient acquisition and respiratory N transformations, supporting increased ammonium release and N mobility. By contrast, microbial communities in lower‐pH soils were enriched in stress‐adaptive functions, which promoted ammonium retention and limited N transformations—thereby diminishing the regulatory influence in N cycling. Together, our findings provide a mechanistic understanding of how microbial community structure and metabolic capabilities regulate ammonium‐related N cycling processes across forests under varying environmental conditions.

## Highlights

- Pedogenic factors influence soil properties, shaping microbial communities and regulating nitrogen cycling and allocation.
- Microbial abundance drives nitrogen cycling rates, while composition and diversity determine ammonium release or retention through metabolic constraints.
- Acidic soils favor stress‐tolerant microbes that retain N, while near‐neutral soils support energy‐efficient taxa that enhance ammonium release and nitrification.

## Graphical abstract

This study reveals how soil pH regulates microbial N allocation in forest soils across Japan. We show that microbial abundance controls the overall rate of N cycling, while microbial composition and metabolic potential determine whether ammonium is released or retained. In acidic soils, stress‐tolerant microbes dominate, promoting N retention and suppressing nitrification. In near‐neutral soils, energy‐oriented taxa are enriched in metabolic pathways for ammonium release and nitrification, enhancing N mobility. These findings highlight a hierarchical structure—from pedogenic factors to soil properties to microbial community structure and function—that governs ecosystem‐level N cycling, with soil pH acting as a key environmental filter.

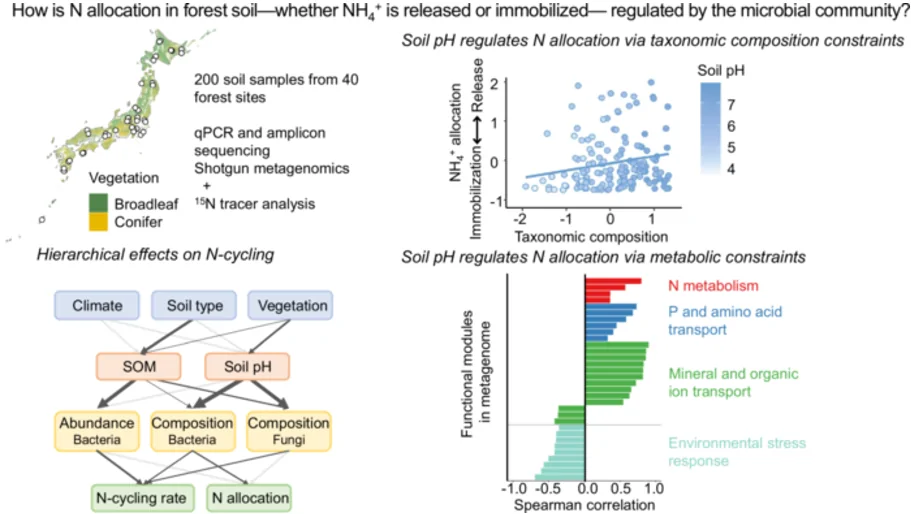

## 1 INTRODUCTION

Soil microbial communities play essential roles in nitrogen (N) cycling processes, such as the mineralization of organic N and the regulation of N availability for plants, which are vital for the productivity of forest ecosystems. As Dokuchaev once stated [1], pedogenic factors such as climate, parent material, and vegetation each give rise to distinct soil properties. These properties, in turn, form a distinctive soil microbial communities that drive N cycling. Building on this understanding, hierarchical relationships—from pedogenic factors to soil properties to microbial communities—can be assumed to underlie variation in N cycling across forests [2, 3].

Among the many processes involved in N cycling, microbial ammonium release (ammonification) and uptake (immobilization) are particularly important [4, 5]. After breaking down organic N into smaller molecules and assimilating them, microbes may release ammonium as a byproduct of metabolism depending on the internal nutrient balance. Conversely, they may take up ammonium in response to the soil N availability and their metabolic demand. These processes constitute major fluxes within the N cycle [6] and strongly influence plant productivity by determining the availability of ammonium to plants [7]. Moreover, they regulate whether N is retained within microbial biomass or directed toward downstream processes such as nitrification [8]. This microbial N allocation affects whether N remains in the soil or is converted into more mobile forms like nitrate, which are more susceptible to leaching and loss, thereby shaping how N is cycled at the ecosystem level [9].

However, the relationship between the structure and function of microbial communities and such microbial N allocation pattern remains poorly understood. This is because both ammonification and ammonium immobilization are fundamental physiological functions that can be carried out by taxonomically and functionally diverse microbes, and are not governed by a limited set of metabolic pathways [10, 11, 12]. Instead, they are integratively regulated by the overall physiological state of the microbial community [4, 5]. As a result, it is difficult to accurately capture these processes using gene‐centric or taxon‐centric approaches that focus on specific functional genes or microbial taxa. This stands in contrast to downstream processes such as nitrification and denitrification, which are controlled by a more specialized set of enzymes and microbial taxa and have therefore been more extensively studied [9, 13, 14, 15]. For example, nitrification is commonly assessed using the marker gene *amoA*, while denitrification is evaluated based on genes such as *nirK*, *nirS*, and *nosZ*, which encode key enzymes involved in the stepwise reduction of nitrate to N gas [9, 13]. Consequently, although soil microbial communities are known to respond sensitively to soil environmental changes [16], it remains unclear how such community‐level shifts influence microbial N allocation pattern.

The biomass or abundance of the entire soil microbial community is often correlated with the rates of “broad processes” [11], such as ammonification, ammonium uptake, and carbon (C) respiration, that are carried out by taxonomically and functionally diverse microbial groups [17, 18, 19]. This observation suggests that microbial N allocation—the balance among ammonification, ammonium uptake, and nitrification—cannot be fully explained by microbial biomass or abundance alone. However, recent studies on C dynamics have shown that the balance between C assimilation for growth and C release is influenced by composition, diversity, and metabolic capabilities of the community [20, 21, 22]. These findings raise the possibility that similar mechanisms may govern microbial N allocation as well [11]. Specifically, microbial communities dominated by taxa with efficient N recycling strategies may exhibit greater N retention than release, compared to those dominated by taxa that tend to excrete N into the environment. Furthermore, changes in community diversity may alter the range of available N transformation pathways, potentially shifting the balance among these microbial processes [23, 24, 25].

Based on this background, we tested the following three hypotheses in this study: (H1) Microbial taxonomic composition and richness influence microbial N allocation, which refers to the balance among ammonification, ammonium uptake, and nitrification; (H2) Because taxonomic composition and diversity are shaped by soil habitat properties, their relationship with N allocation is also dependent on the environmental context; and (H3) Despite such a context‐dependent relationship, microbial N allocation is ultimately determined by the metabolic capabilities of the entire microbial community. Therefore, if these capabilities are shaped by taxonomic composition, a causal pathway from taxonomic composition to metabolic capabilities to N allocation can be expected.

**Figure 1:**
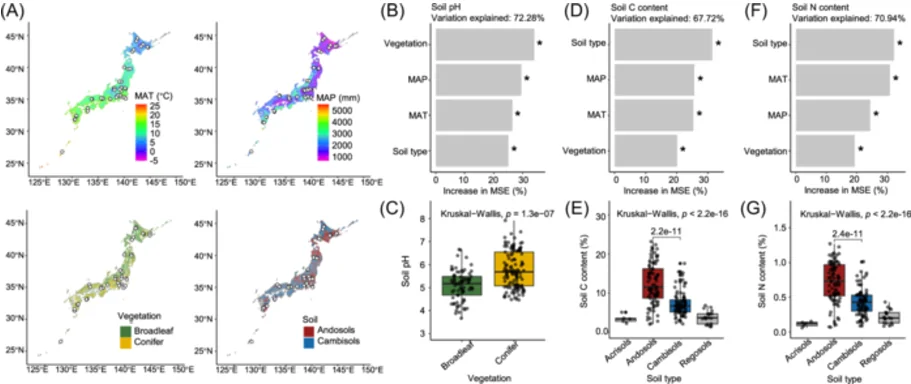
Pedogenic factors shaping soil habitat properties. (A) The map displays soil sampling locations across Japan, illustrating the distributions of mean annual temperature (MAT), mean annual precipitation (MAP), vegetation (broadleaved and coniferous forests), and soil types (Andosols and Cambisols). The soil samples were collected from 40 sites and represented a variety of pedogenic factors. (B−G) The panels demonstrate the mean predictor importance, as indicated by an increase in mean squared error (MSE), of various pedogenic factors and the most influential predictor on soil pH in (B) and (C), the soil carbon content in (D) and (E), and the soil N content in (F) and (G). The asterisks in Panels B, D, and F represent statistical significance (*p* < 0.05). Differences in soil pH among vegetations and in soil C and N among soil types were evaluated using the Kruskal–Wallis test.

To empirically test these hypotheses, we investigated the hierarchical effects of pedogenic factors (climate, soil type derived from different parent materials, and vegetation) and soil habitat properties (soil C and N contents and pH) on N cycling rates and N allocation, as mediated by microbial community components (microbial abundance, taxonomic composition, and taxonomic richness) across 40 forests in Japan. Specifically, we assessed these components of bacterial and fungal communities using 16S rRNA gene (for bacteria) and the Internal Transcribed Spacerregion (for fungi) sequencing and quantitative assays. We quantified overall N cycling rates by measuring the gross rates of key processes, including ammonification, nitrification, and the immobilization of ammonium or nitrate, through ^15^N tracer experiments. We assessed N allocation by evaluating the microbial community's capacity for N retention or release, as indicated by two key ratios: the ratio of ammonification (ammonium release) to ammonium immobilization (ammonium uptake) and the ratio of nitrification to ammonification. A higher ratio indicates greater N release from the microbial community [6, 26, 27]. In the former case, this reflects a tendency for the microbial community to release more N than it retains. In the latter case, the ratio reflects the proportion of ammonium that is further oxidized to nitrate, a more mobile form of N, thereby indicating a shift toward enhanced N mobility and a greater potential for N loss via leaching. We also analyzed the genetic diversity that mediates N transformation processes and the overall metabolic capabilities of the community via shotgun metagenomics. Our large‐scale investigation covered 40 unique forest sites across a climate gradient in Japan, selected for their diverse and balanced representations of climate, soil types, and vegetation (Figure 1A). The climate ranges from cool temperate to warm temperate and subtropical. The soil types investigated included Cambisols (17 sites), Andosols (18 sites), Regosols (4 sites), and Acrisols (1 site). The vegetation types studied encompassed broadleaved forests (15 sites) and coniferous forests (25 sites). Cambisols are derived from weathered sedimentary rocks, while Andosols form from volcanic ash. Regosols originate from recent sediment deposition, and Acrisols are associated with weathered tropical soils. This selection enabled in‐depth analysis of the context‐dependent nature of microbial community response and its N cycling outcomes.

## 2 RESULTS

### Hierarchical effects of pedogenic factors, soil habitat properties, and microbial community components on N cycling

We examined how pedogenic factors and soil habitat properties hierarchically influence N cycling rates and N allocation via microbial community components across all forest sites. The ranges of measured variables are summarized in Tables S1 and S2. Notably, soil pH ranged from 3.7 to 7.9. Gross ammonification rates (mean: 3.88 mg N kg^−1^ soil day^−1^) were substantially higher than gross nitrification rates (mean: 0.94 mg N kg^−1^ soil day^−1^). On average, 36% of the ammonium produced via ammonification was utilized in nitrification, while 55% was incorporated into microbial biomass through immobilization.

Pedogenic factors (climate, soil type, and vegetation) exerted deterministic effects on soil habitat properties (C and N contents and pH) (Figure 1B−G). Vegetation was the primary determinant of soil pH, with additional influences from mean annual precipitation (MAP) and mean annual temperature (MAT) (Figure 1B); coniferous forests exhibited significantly higher pH than broadleaved forests (Kruskal−Wallis, *p* < 0.01) (Figure 1C). In contrast, soil type was the primary determinant of soil C and N contents, with additional influences from MAT and MAP (Figure 1D,F); Andosols presented the highest soil C and N contents, followed by Cambisols, whereas Regosols and Acrisols presented lower C and N contents (Kruskal−Wallis, *p* < 0.01) (Figure 1E,G).

**Figure 2:**
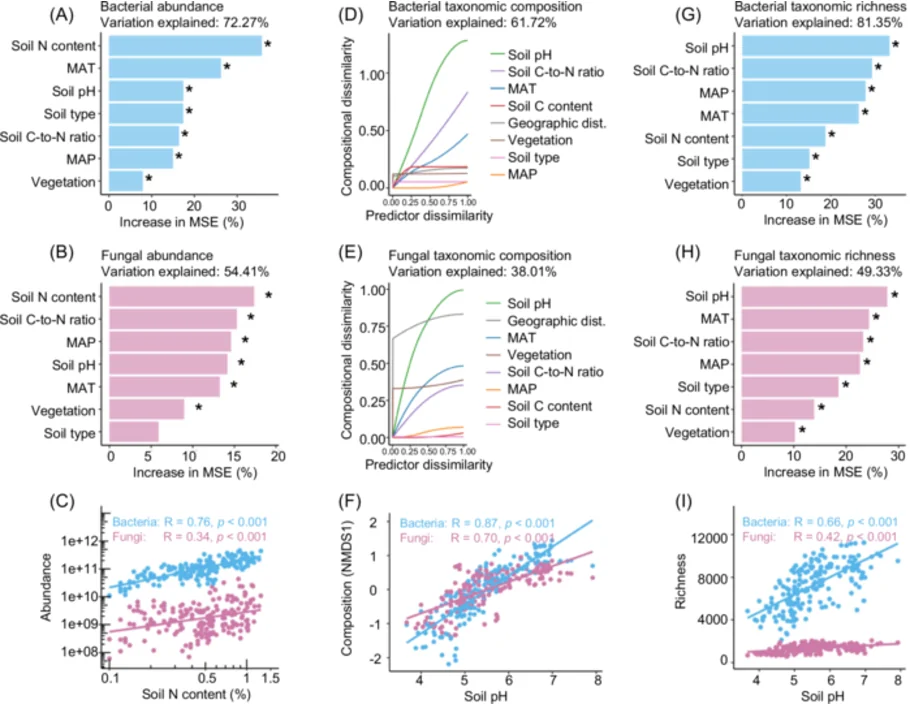
Pedogenic factors and soil habitat properties shaping the soil microbiome. (A−C) The panels demonstrate the mean predictor importance, as indicated by an increase in increase in mean squared error (MSE), of various pedogenic factors and soil habitat properties and the most influential predictor of bacterial (blue) and fungal (pink) abundances. (D−F) The panels demonstrate the mean predictor importance of various pedogenic factors and soil habitat properties on the basis of the generalized dissimilarity model (GDM) and the most influential predictor of bacterial (blue) and fungal (pink) community compositions. Taxonomic composition was measured via Bray‒Curtis dissimilarity for both bacterial and fungal communities. For the correlation analysis with soil pH, community composition is represented by axis values from nonmetric multidimensional scaling (NMDS) ordination plots for bacterial or fungal communities. (G−I) The panels demonstrate the mean predictor importance of various pedogenic factors and soil habitat properties, and the most influential predictor of bacterial (blue) and fungal (pink) amplicon sequence variant (ASV) richness. The asterisks in (A, B, G and H) represent statistical significance (*p* < 0.05).

Soil habitat properties had stronger effects on microbial community components (abundance, taxonomic composition, and taxonomic richness) than pedogenic factors, with bacterial communities being more fully explained than fungal communities (Figure 2A–I). Soil N content was the primary determinant of microbial abundance, alongside MAT for bacteria and the soil C‐to‐N ratio for fungi (Figure 2A,B). Both bacterial and fungal abundances increased significantly with higher soil N (single regression, *p* < 0.01) (Figure 2C). Soil pH emerged as the dominant factor shaping taxonomic composition for both bacteria and fungi, as revealed by generalized dissimilarity modeling (GDM) analysis. The model explained 61.72% of the variation in bacterial composition and 38.01% in fungal composition (Figure 2D,E). Nonmetric multidimensional scaling (NMDS) and permutational multivariate ANOVA (PERMANOVA) confirmed the strong effect of pH (*p* < 0.01) (Figure S1), with NMDS axis 1 highly correlated with soil pH (single regression, *p* < 0.01) (Figure 2F). Notably, microbial taxa whose relative abundances increased in higher pH soils include the order *Rhizobiales* and *Burkholderiales* (phylum *Proteobacteria*), *Chthoniobacterales* (*Verrucomicrobiota*), *Vicinamibacterales* (*Acidobacteriota*), *Mortierellales* (*Mortierellomycota*), *Sebacinales* (*Basidiomycota*), and *Sordariales* (*Ascomycota*) (Figure S2 and Table S3). In contrast, microbial taxa whose relative abundances increased in lower pH soils include the order *Acidobacteriales* and *Subgroup 2* (phylum *Acidobacteriota*), *Elsterales* (*Proteobacteria*), *Gemmatales* (*Planctomycetota*), *Frankiales* (*Actinobacteriota*), *Pedosphaerales* (*Verrucomicrobiota*), *Helotiales* and *Chaetothyriales* (*Ascomycota*), and *Russulales* (*Basidiomycota*) (Figure S2 and Table S3). Additionally, the relative abundances of the order *Nitrospirales* (phylum *Nitrospirota*) and *Nitrosococcales* (*Proteobacteria*) increased significantly with soil pH, with *Nitrospirales* typically involved in nitrite oxidation or complete ammonia oxidation (comammox), and *Nitrosococcales* known for their role as ammonia‐oxidizing bacteria. Soil pH was also the primary determinant of bacterial and fungal taxonomic richness, with additional contributions from the soil C‐to‐N ratio for bacteria and MAT for fungi (Figure 2G,H). Both bacterial and fungal richness increased significantly with higher soil pH (single regression, *p* < 0.01) (Figure 2I).

**Figure 3:**
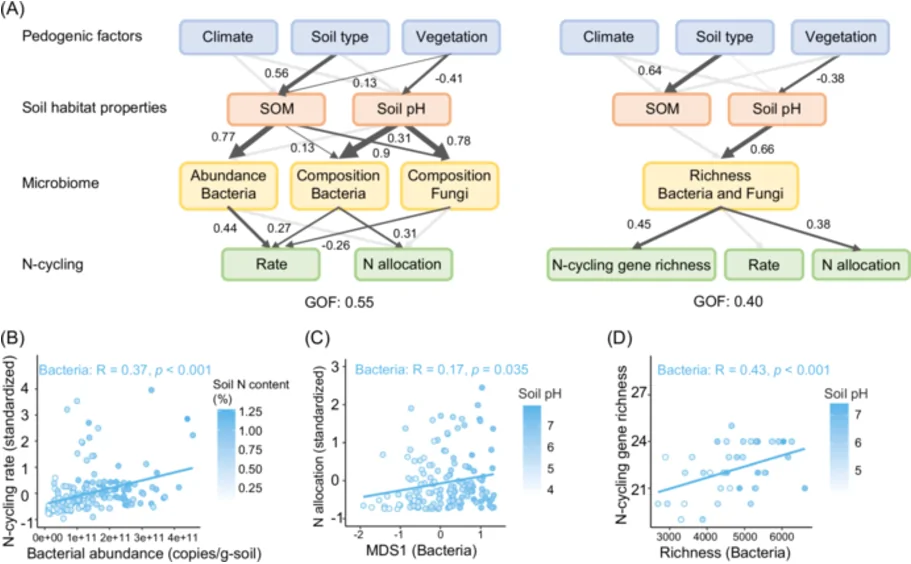
Hierarchical effects of pedogenic factors, soil habitat properties, and microbial community components on N cycling. (A) The results of the PLS‒PM analysis reveal a cascading relationship from pedogenic factors through soil habitat properties to the microbial community and N cycling. Taxonomic composition is represented by axis values from nonmetric multidimensional scaling (NMDS) ordination plots for both bacterial and fungal communities. The N cycling rate is conceptualized as a latent variable reflecting the gross rates of ammonification, nitrification, and ammonium or nitrate consumption, with higher values indicating increased rates. N allocation, which is also a latent variable, comprises the ratio of nitrification‐to‐ammonification and the ratio of ammonification (as ammonium release)‐to‐ammonium immobilization (as uptake), with higher values suggesting greater N release from the soil microbial community. N cycling gene richness is measured by the richness of N‐cycling genes in metagenomes. The numbers next to the arrows represent the path coefficients, which indicate the strength and direction of the linear relationships between variables. Dark gray arrows signify significant relationships (*p* < 0.05), while light gray arrows indicate nonsignificant relationships (*p* > 0.05). The models are assessed using the goodness‐of‐fit statistic: 0.55 (*n* = 190, left) and 0.40 (*n* = 38, right). The width of each arrow corresponds to the strength of the path coefficients. The panels show the microbial community components with the strongest effects on N cycling, demonstrating the relationships between bacterial abundance and the N cycling rate in (B), bacterial taxonomic composition and N allocation in (C), and bacterial taxonomic richness and N cycling gene richness in (D). GOF, goodness of fit; N, Nitrogen; SOM, soil organic matter.

Finally, we used partial least squares path modeling (PLS‐PM) to clarify the hierarchical structure of relationships (Figure 3A and Table S4). Bacterial abundance was the primary determinant of the overall N cycling rate, a latent variable defined by the gross rates of ammonification, nitrification, and ammonium/nitrate consumption (Figure 3A). These rates were also influenced, though to a lesser extent, by bacterial and fungal taxonomic compositions. Notably, N cycling rates increased significantly with increasing bacterial abundance (single regression, *p* < 0.01) (Figure 3B). In contrast, bacterial composition was the sole determinant of N allocation, which was represented by two ratios: ammonification (ammonium release) to ammonium immobilization (uptake), and nitrification to ammonification (Figure 3A). While these ratios capture different stages of microbial N transformations, they were significantly positively correlated (Table S5), suggesting a common underlying gradient in microbial N release potential. Therefore, we defined a latent variable for N allocation that integrates both ratios to capture the overall tendency of the microbial community to promote N release. Taxonomic composition in higher‐pH soils was associated with higher ratios, indicating greater N release from the microbial community and increased nitrification (single regression, *p* < 0.05) (Figure 3C). To stabilize the model, we excluded fungal abundance due to its high collinearity with bacterial abundance, and taxonomic richness due to its collinearity with taxonomic composition. Instead, we assessed taxonomic richness independently. Taxonomic richness, a latent variable defined by bacterial and fungal taxonomic richness, was a key determinant of both the richness of N‐cycling genes identified from shotgun metagenomic analysis (one replicate per site across 40 forests) and the N allocation (Figure 3A and Table S6). Notably, greater taxonomic richness in higher‐pH soils was significantly associated with increased richness of N‐cycling genes (single regression, *p* < 0.01) (Figure 3D) and with higher N allocation ratios—indicating greater N release and enhanced nitrification (Figure 3A and Table S7). These PLS‐PM‐based hierarchical relationships were consistent with, and strongly supported by, correlation analyses among individual variables (Spearman correlation) (Table S5). Both approaches indicated that microbial community components exerted a stronger influence on N cycling than pedogenic factors or soil habitat properties.

### Context‐dependent effects of microbial community components on N allocation

To test the hypothesis that the relationship between microbial community components and N cycling facets varies across environmental conditions, we stratified the data set by vegetation type and soil type—two major drivers of soil pH, as well as soil C and N contents—and examined the respective roles of microbial abundance and taxonomic composition in regulating N cycling outcomes.

**Figure 4:**
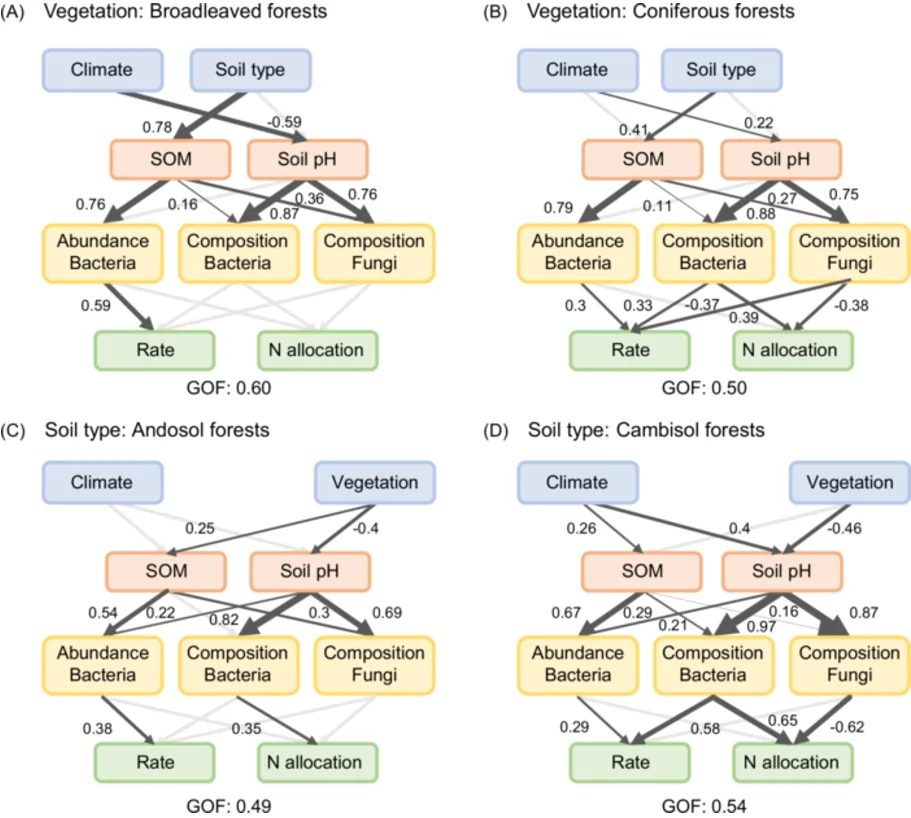
Context dependency of microbial community responses and their N cycling outcomes. The panels show the relationship between microbial community response and N cycling outcome, as observed within broadleaved forests and coniferous forests (A, B), as well as Andosol forests and Cambisol forests (C, D). The numbers next to the arrows represent the path coefficients, which indicate the strength and direction of the linear relationships between variables. Dark gray arrows signify significant relationships (*p* < 0.05), while light gray arrows indicate nonsignificant relationships (*p* > 0.05). The width of each arrow corresponds to the strength of the path coefficients. The models are assessed using the goodness‐of‐fit statistic: 0.60 (*n* = 65) in broadleaved forests, 0.50 (*n* = 125) in coniferous forests, 0.49 (*n* = 90) in Andosol forests, and 0.54 (*n* = 85) in Cambisol forests. GOF, Goodness of Fit; N, Nitrogen; SOM, Soil Organic Matter.

We first stratified the data by vegetation type (broadleaved vs. coniferous forests; Figure 4A,B). These forest types harbored distinct microbial communities, shaped primarily by differences in soil pH—broadleaved forests being more acidic (mean pH = 5.09, range = 3.67–6.67) and coniferous forests closer to neutral (mean pH = 5.77, range = 4.47–7.92) (Figures 1C and 2F). In broadleaved forests, bacterial abundance alone influenced the N cycling rate, whereas taxonomic composition had no significant effect on either N cycling rate or N allocation (Figure 4A and Table S8). In contrast, in coniferous forests, taxonomic composition (both bacterial and fungal) contributed as much as abundance to N cycling rate, and played a dominant role in shaping N allocation (Figure 4B and Table S9).

We next stratified the data by soil type (Andosols vs. Cambisols; Figure 4C,D). Andosol forests, with higher soil C and N contents (mean N content = 0.71%, range = 0.07%–1.27%), supported greater bacterial abundance compared to Cambisol forests (mean N content = 0.43%, range = 0.09%–1.01%) (Figures 1E,G and 2C, Table S5). In Andosol forests, bacterial abundance explained the N cycling rate, while bacterial taxonomic composition—though unrelated to the rate—was a key determinant of N allocation (Figure 4C and Table S10). In Cambisol forests, where bacterial abundance was lower, taxonomic composition exerted a stronger influence on both N cycling rate and allocation (Figure 4D and Table S11).

### Metabolic capabilities constraining context dependence

We characterized microbial metabolic capabilities by the composition and abundance of Kyoto Encyclopedia of Genes and Genomes (KEGG) metabolic modules. Using shotgun metagenomic data, we tested whether the context‐dependent effects of taxonomic composition on N cycling—particularly the balance among ammonification, ammonium uptake, and nitrification—were mediated by these community‐level metabolic capabilities. Specifically, we examined whether shifts in taxonomic composition resulted in corresponding change in metabolic capabilities, which in turn affected N allocation.

**Figure 5:**
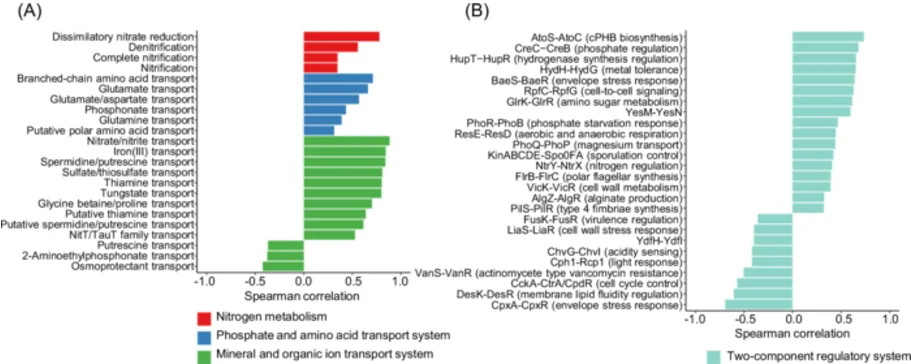
Changes in the metabolic capabilities of the microbial community in response to changes in the soil pH. The panels show the Spearman correlation (*p* < 0.05) between the abundance of Kyoto Encyclopedia of Genes and Genomes (KEGG) modules and soil pH. Modules with a positive value are more abundant at higher pH values, whereas those with a negative value are more abundant at lower pH values. (A) KEGG modules involved in mineral and organic ion transport systems, Phosphate and amino acid transport systems, and Nitrogen metabolism. (B) KEGG modules involved in the Two‐component regulatory system.

Metagenomic analysis revealed that taxonomic compositional and metabolic shifts occurred concurrently, as the composition was strongly correlated with metabolic capabilities, and both closely aligned with variations in soil pH (PERMANOVA *p* < 0.01; Pearson correlation *p* < 0.01) (Figure S3). We therefore examined which metabolic functions are sensitive to a change in soil pH, with a particular focus on the functions related to N cycling (Figure 5A,B). We specifically analyzed KEGG modules associated with energy metabolism and environmental information processing, including N metabolism, nutrient transport (phosphate, amino acids, minerals, and organic ions), and the two‐component regulatory system (Table S2). Among the pH‐correlated modules (Spearman correlation *p* < 0.05), those involved in dissimilatory N metabolism, including dissimilatory nitrate reduction (nitrate → nitrite → ammonium), denitrification (nitrate → nitrite → NO → N_2_O → N_2_), complete nitrification (ammonia → nitrite → nitrate), and nitrification (ammonia → nitrite), consistently showed positive correlations with pH (Figure 5A). Likewise, modules involved in phosphate and amino acid transport (e.g., branched‐chain amino acid and glutamate transporters) also showed positive correlations. Most mineral and organic ion transport modules, including nitrate and nitrite transport, were positively correlated with pH as well, although some—such as osmoprotectant transport—showed negative correlations (Figure 5A). The two‐component regulatory system displayed both positive and negative correlations (Figure 5B). Positive correlations included modules involved in aerobic and anaerobic respiration (e.g., ResE–ResD) and N regulation (NtrY–NtrX). In contrast, negative correlations were observed in stress response systems, such as envelope stress (CpxA–CpxR), membrane lipid fluidity (DesK–DesR), acidity sensing (ChvG–ChvI), and cell wall stress (LiaS–LiaR).

## 3 DISCUSSION

Among the many processes involved in N cycling, microbial ammonification and immobilization are particularly important, as they constitute major fluxes and directly influence N retention, loss, and plant productivity. However, because these processes are shared across diverse microbial taxa and involve a variety of metabolic pathways, they are challenging to assess using gene‐ or taxon‐specific approaches. To address this, we examined how the abundance, taxonomic composition, taxonomic richness, and metabolic capabilities of entire microbial communities relate to these ammonium‐related processes, providing an ecosystem‐level understanding of microbial regulation of ammonium release, uptake, and nitrification.

We first revealed a hierarchical structure in which pedogenic factors and soil habitat properties influence N cycling rates and N allocation via microbial community components across forests (Figure 3A). Microbial abundance increased with higher soil N content and functioned as the primary driver of overall N cycling rates. In contrast, the taxonomic composition—that is, the dominance of specific taxa—had a secondary influence on N cycling rates. This relationship between microbial abundance and N transformation rates is consistent with previous global studies reporting that gross ammonification and N immobilization are tightly coupled with soil microbial biomass and soil N content [17, 18], and that net N mineralization also correlates with soil microbial biomass or soil N content [19, 28]. Moreover, both taxonomic composition and richness were strongly shaped by soil pH, and these patterns were reflected in microbial N allocation (Figure 3A), thus supporting Hypothesis 1. Taxonomic compositions assembled in lower‐pH soils tended to favor ammonium uptake and retention, whereas those in higher‐pH soils promoted ammonium release and nitrification, resulting in increased N mobility (Figure 3C). Microbial communities with greater taxonomic richness also exhibited enhanced N release, likely because they were capable of utilizing a broader range of N transformation pathways (Figure 3A,D) [23, 24, 25]. However, the effects of taxonomic composition and diversity may be interdependent; near‐neutral soils supported taxonomic compositions that allowed a wider variety of taxa to coexist, thereby expanding the functional gene repertoire involved in N cycling and promoting greater N mobility in soil.

Microbial taxa with greater relative abundance in higher pH soils (Figure S2 and Table S3) were reported to exhibit neutrophilic strategies or are frequently detected in neutral soils [29, 30, 31, 32, 33]. These microbes could possess efficient nutrient acquisition mechanisms, including N fixation (*Rhizobiales*), rapid decomposition and mineralization of organic matter (*Mortierellales*, *Sordariales*), nitrification (*Nitrospirales*, *Nitrosococcales*), symbiotic nutrient exchange (*Sebacinales*, and some *Burkholderiales* and *Rhizobiales*), and the utilization of simple and complex substrates (*Chthoniobacterales*) [29, 30, 31, 32, 33]. In contrast, microbial taxa with greater relative abundance in lower pH soils (Figure S2 and Table S3) were reported to exhibit acidophilic strategies or are consistently detected under acidic conditions [34, 35, 36, 37, 38]. These microbes could be typically associated with oligotrophic strategies, characterized by slow growth and resource‐efficient life histories (*Acidobacteriales*, *Subgroup 2*), metabolic versatility under low pH (*Frankiales*, *Gemmatales*, *Pedosphaerales*), organic nitrogen acquisition through mycorrhizal symbiosis (*Helotiales*, *Russulales*), and high tolerance to environmental stress (*Chaetothyriales*) [34, 35, 36, 37, 38, 39]. The ecological traits of these microbial taxa appear to be consistent with the patterns of nitrogen allocation observed across soil pH gradients, as measured in our study.

Next, we demonstrated that the regulatory effects of microbial abundance and taxonomic composition on N cycling were highly dependent on environmental context (Figure 4A–D), thereby supporting Hypothesis 2. This context dependency was governed by vegetation type (broadleaved vs. coniferous forests) and soil type (Andosol forests vs. Cambisol forests), which determined key soil habitat properties—soil pH and N content, respectively. In coniferous forests with soils closer to neutral pH, taxonomic composition had a substantial influence on both N cycling rates and N allocation. In contrast, in broadleaved forests with lower pH soils, taxonomic composition had no effect on these outcomes, while bacterial abundance still significantly affected N cycling rates. We further found that in Andosol forests, where soil N content and microbial abundance were higher, bacterial abundance was the dominant factor controlling N cycling rates. Conversely, in Cambisol forests, where soil N content and microbial abundance were lower, the effect of taxonomic composition became more pronounced. Notably, the taxonomic composition of bacterial community consistently regulated N allocation across both soil types, with particularly strong effects observed in Cambisols. Together, these findings highlight two forms of environmental context dependency: (1) the suppression of taxonomic composition‐based regulation under lower pH conditions, and (2) a shift between abundance‐driven and composition‐driven regulation, depending on soil N content and microbial abundance. This context‐dependent microbial regulation may help explain why microbial abundance often predicts N process rates at broad spatial scales [17, 18, 19], but not necessarily at local scales [14, 40].

Finally, although the extent to which taxonomic composition regulates the balance between ammonium release, uptake, and subsequent nitrification was found to be dependent on soil pH, we showed that such N allocation is ultimately constrained by the overall metabolic capabilities of the microbial community (Figure 5A,B), which are shaped by its taxonomic composition (Figure S3)—supporting Hypothesis 3. Shotgun metagenomic analysis revealed that pH‐driven shifts in taxonomic composition led to corresponding changes in the metabolic capabilities of the entire microbial community. Such coordinated changes have also been reported in previous studies [41]. Furthermore, many of the metabolic modules that responded to soil pH change were strongly correlated with each other (Figure S4), suggesting that instead of acting independently, a suite of modules co‐varied in a coordinated manner, restructuring the community's overall metabolic capabilities.

In more neutral soils, modules involved in nutrient transport and acquisition (such as the uptake of amino acids, phosphate, and iron) as well as modules related to aerobic and anaerobic respiration and to both respiratory and regulatory N metabolism were enriched (Figure 5A,B). These included specific KEGG modules such as nitrification (M00528 and M00804; ammonia → nitrite → nitrate) and denitrification (M00529; nitrate → nitrite → NO → N_2_O → N_2_). Nitrification promotes the accumulation of nitrate, thereby enhancing N mobility, while denitrification facilitates N loss via gaseous emissions. This pattern suggests the selective enrichment of taxa with energy‐generating metabolic strategies and active N transformation capabilities. These findings were consistent with our direct measurements, which showed increased ammonium release and nitrification relative to ammonium uptake in these soils, resulting in enhanced N mobility. In contrast, in lower‐pH soils, modules associated with membrane stability, acidity sensing, and cellular protection against environmental stress were enriched (Figure 5B). This pattern indicates the selective enrichment of taxa with protective strategies that confer resistance to acidic environments. Acidic environments impose high proton concentrations around the outer cell membrane, requiring microbes to employ pH‐responsive transporters and enzymes to maintain macromolecular integrity and metabolic function [42, 43]. These stress responses are energetically costly, necessitating the allocation of substantial energy toward cellular protection and thereby limiting the resources available for N metabolism [21, 44]. This metabolic shift toward stress adaptation was consistent with our direct measurements, which showed that ammonium uptake and retention were enhanced in acidic soils. It also provided a mechanistic explanation for why the influence of taxonomic composition on N cycling was diminished in lower pH soils.

While it is well known that microbial taxonomic composition and richness are particularly sensitive to soil pH [45], how such shifts influence “broad” N transformation processes remained unclear. As shown by our findings and previous studies [3, 40], microbial abundance can be a stronger driver of process rates than changes in taxonomic composition and richness, meaning that shifts in composition and richness do not necessarily lead to changes in process rates [3, 10]. By testing Hypotheses 1−3, that changes in taxonomic composition and richness alter the overall metabolic capabilities of the community and consequently affect microbial N allocation—specifically whether ammonium is retained within microbial biomass or released into more mobile forms—we clarified the role of microbial taxonomic composition and richness in N cycling. This focus is important for understanding ecosystem‐level nitrogen cycling, as microbial nitrogen allocation directly influences forest productivity and the risk of nitrogen loss [12, 46]. Forest ecosystems often maintain closed nitrogen cycles through microbial retention, but the degree of closure varies with vegetation type and forest age [47, 48, 49].

Additionally, microbial community‐level metabolic shifts have gained increasing attention in recent studies on soil C dynamics [20, 44, 50, 51]. These studies highlighted how microbial communities allocated assimilated C, either toward biomass production or toward other functions such as stress responses, enzyme secretion, and CO_2_ release. In acidic soils, a greater proportion of C was allocated to mitigate acid stress, whereas in near‐neutral soils, more C was allocated to biomass production [20, 21]. Because biomass formation requires energy, efficient functioning of both dissimilatory and assimilatory N metabolism may be necessary to support microbial growth under these conditions. Our findings were consistent with these observations on microbial C use and suggested that pH‐driven changes in microbial metabolic capabilities influenced not only C allocation strategies but also N use strategies. Moving forward, an integrated framework is needed to understand how microbial communities allocate assimilated C and N, which shape ecosystem C and N cycling.

## 4 CONCLUSION

This study reveals how ammonium is allocated by microbial communities—either retained within microbial biomass or transformed into more mobile forms such as nitrate—shaped by the community's taxonomic composition and metabolic capabilities. By studying forest ecosystems across Japan, we identified a hierarchical structure in which pedogenic factors influence soil properties, which in turn shape microbial communities and ultimately regulate both the rate of N cycling and patterns of N allocation. Among these soil properties, soil pH played a pivotal role in microbial regulation of N allocation, through shifts in the community's taxonomic composition and metabolic capabilities. In acidic soils, pH‐induced stress favored the dominance of stress‐tolerant taxa with lower N transformation activity, promoting N retention. In contrast, near‐neutral soils supported energy‐oriented taxa capable of driving dissimilatory N transformations such as ammonium release and nitrification, thereby enhancing N mobility. These findings underscore the role of microbial taxonomic composition and metabolic capabilities in regulating N allocation and highlight soil pH as a key environmental filter linking soil conditions to ecosystem‐level N cycling.

## 5 METHODS

### Study sites and sampling

The datasets used in this study covered 40 forest sites across Japan (Figure 1A). The latitude ranged from 44°22' N to 26°45' N. The longitude ranged from 144°39' E to 128°13' E. The MAT ranged from 4.4°C to 20.9°C, and the MAP ranged from 820 to 3080 mm. The climatic zones for most sites were temperate, with one site being subtropical. The study sites included 15 broadleaved forests consisting of Japanese oak (*Quercus crispula*), Japanese beech (*Fagus crenata*), Konara oak (*Quercus serrata*), *Ilex pedunculosa*, *Castanopsis sieboldii*, *Quercus acutissima*, and *Tilia maximowicziana*. The other 25 sites were coniferous forests of Japanese cedar (*Cryptomeria japonica*), cypress (*Chamaecyparis obtusa*), larch (*Larix kaempferi*), red pine (*Pinus densiflora*), *Abies sachalinensis*, and *Abies firma*. The soil types were classified into Cambisols (17 sites), Andosols (18 sites), Regosols (4 sites), and Acrisols (1 site) (Table S1). We conducted sampling once at each forest site, either in summer or autumn, avoiding periods of extreme soil moisture conditions, such as the rainy season (early summer). We collected mineral soil from 0 to 10 cm at five locations at each forest site as described previously [52].

### Soil chemical analyses and gross N transformation rate measurements

We measured the C and N contents of mineral soils using a Carbon–Nitrogen analyzer after air‐drying and grinding the samples. We also measured the soil pH (H_2_O) after water extraction. For the N cycling rate and N allocation, we measured the gross N transformation rates in the soil using the ^15^N isotope dilution method [53]. The measurements were performed as previously described [3, 54]. Gross ammonification, ammonium consumption, nitrification, and nitrate consumption rates were calculated according to the methods described by Hart et al. [53]. The gross ammonium immobilization or uptake rate was calculated as ammonification minus nitrification [55].

### rRNA gene quantification and sequencing

We analyzed the abundance of bacterial and fungal communities by rRNA gene quantification, using 200 samples with five replicates from 40 forests. The DNA was extracted from 0.4 g of the soil and purified. The prokaryotic 16S rRNA gene and fungal 18S rRNA gene were quantified by quantitative polymerase chain reaction to estimate bacterial and fungal abundances. We also analyzed the taxonomic composition and richness of the bacterial and fungal communities via rRNA gene sequencing (200 samples with 5 replicates from 40 forests). The prokaryotic 16S rRNA genes and fungal ITS genes (ITS2 region) were sequenced on an Illumina MiSeq platform and analyzed to estimate the composition and richness of the bacterial and fungal communities. The 16S rRNA gene and ITS2 region sequencing data obtained via MiSeq were processed using UPARSE [56], VSEARCH (v2.22.1) [57], and QIIME2 (v2023.2) [58]. Additional details are available in the Supplementary information.

### Shotgun metagenomic sequencing and analysis

We analyzed the metabolic capabilities of the microbial community using shotgun metagenomic sequencing. In this study, we characterized microbial metabolic capabilities by using the composition and abundance of the KEGG functional modules, which represent the community‐level potential to perform specific metabolic functions. For metagenomic analysis, one replicate from each site was selected for sequencing (40 samples, 1 sample from each forest). Shotgun libraries were prepared and sequenced on an Illumina HiSeq platform with 251 bp paired‐end reads. Three million amino acid sequences per sample were mapped to functional modules in KEGG, and the module abundance was calculated (Table S2) via Genome Metabolic and Physiological Potential Evaluator (Genomaple) [59, 60]. To allow for cross‐sample comparisons, module abundances were normalized using the abundance of a ribosomal module (M90000), which consists of 79 ribosomal proteins corresponding to 31 KOs shared between bacteria and archaea. Genes involved in the “nitrogen metabolism” pathway (M00175, M00531, M00530, M00529, M00528, M00804, and M00973) assigned via KEGG Orthology (KO) were utilized to define N‐cycling KO genes (Table S6). Additional details are available in the Supplementary information.

### Statistical analyses

All the statistical analyses were performed using R (v4.3.2) [61]. We conducted classification random forest analyses to explore the relative importance of pedogenic factors in shaping soil habitat properties. In our models, different pedogenic factors (MAP, MAT, vegetation, and soil type) were included as predictors, and soil habitat properties (soil pH and soil C and N contents) were the response variables. Next, we conducted random forest analyses to explore the relative importance of pedogenic factors and soil habitat properties in shaping the abundance and richness of bacterial and fungal communities. Additionally, we performed a linear correlation analysis between the most significant predictor and the response variables.

We conducted generalized dissimilarity modeling [62] to explore the relative contributions of pedogenic factors and soil habitat properties to the taxonomic compositions of the bacterial and fungal communities. We calculated the dissimilarity of composition using the Bray–Curtis index as a distance matrix. The predictors included MAT, MAP, vegetation, soil type, soil pH, soil C and N contents, soil C‐to‐N ratio, and the geographical distance between sites. We further conducted NMDS using the Bray–Curtis distance matrix to explore the similarity of the bacterial and fungal taxonomic compositions. Because GDM analysis identified soil pH as the most significant predictor of both bacterial and fungal community compositions, we used permutational multivariate ANOVA (PERMANOVA) to determine whether soil pH explained the variation in composition across samples. Additionally, we performed a linear correlation analysis between the soil pH and the NMDS1 scores.

We used partial least squares path modeling (PLS−PM) [63] to examine the hierarchical effects of pedogenic factors, soil habitat properties, and soil microbial community components on N cycling. For the abundance and taxonomic composition, we used 5 replicates per site. For taxonomic richness, we used 1 replicate per site because we specifically asked whether greater microbial richness leads to greater richness of N‐cycling genes. We used both manifest variables and latent variables to test our hypothesis. The overall N cycling rate was conceptualized as a latent variable reflecting gross rates of ammonification, nitrification, and ammonium or nitrate consumption, where higher values indicated greater rates. N allocation was also represented by two ratios: the nitrification‐to‐ammonification ratio and the ratio of ammonification (ammonium release) to ammonium immobilization (ammonium uptake). Because these two ratios were significantly positively correlated (Table S5), we defined a latent variable for N allocation that integrates both ratios to capture the overall tendency of the microbial community to promote N release, with higher values indicating greater N release from the soil microbial community or potentially from the ecosystem. The significance of the path coefficients was assessed using bootstrapping with 1000 resamples, and *p* < 0.05 were considered statistically significant. The goodness‐of‐fit index was used to evaluate the overall quality of the PLS‐PM model, calculated as the square root of the product of the average *R*² of endogenous latent variables and the average variance extracted (AVE) of all latent variables. The goodness‐of‐fit value in our model ranged from 0.38 to 0.60, exceeding the commonly accepted threshold of 0.36 for strong fit [64].

For the metagenomic data, we calculated the dissimilarity of the functional module composition and taxonomic composition using the Bray–Curtis distance and used PERMANOVA to determine whether the soil pH explained the variation in the compositions across samples. Additionally, we performed a linear correlation analysis between the NMDS1 scores of the taxonomic composition and the NMDS2 scores of the functional module composition. We also performed a correlation analysis by calculating Spearman's rank correlation coefficient between the abundance of each functional module and the soil pH. A full description of the methods is provided in the Supplementary Information.

## AUTHOR CONTRIBUTIONS

**Yaping Liu**: Investigation; writing—review & editing; formal analysis; methodology, validation; visualization. **Yuta Ise:** Investigation; writing—review & editing; formal analysis; methodology; validation; visualization. **Hideto Takami**: Methodology; validation; writing—review & editing; software. **Rieko Urakawa**: Writing—review & editing, data curation, resources. **Ryunosuke Tateno**: Writing—review & editing; data curation; resources. **Atsushi Toyoda**: Writing—review & editing; methodology; software. **Nobuhito Ohte**: Writing—review & editing; funding acquisition; data curation; resources. **Weiyu Shi**: Writing—review & editing. **Lin Jiang**: Writing—review & editing. **Kazuo Isobe**: Conceptualization; investigation; funding acquisition; writing—original draft; methodology, validation; visualization; writing—review & editing; project administration; resources; supervision; data curation; formal analysis.

## CONFLICT OF INTEREST STATEMENT

The authors declare no conflicts of interest.

## 1 ETHICS STATEMENT

No animals or humans were involved in this study.

## Supporting information


**Figure S1.** Taxonomic composition shifts in response to soil habitat pH.
**Figure S2.** Shifts in taxonomic composition of bacterial and fungal communities at the order level in response to soil pH.
**Figure S3.** Shifts in taxonomic and metabolic composition in response to soil pH.
**Figure S4.** Spearman correlations among KEGG modules that were significantly associated with soil pH.

**Table S1.** The range of pedogenic factors, soil habitat properties, microbiome components, N cycling, and shotgun metagenomic results.
**Table S2.** KEGG modules associated with N metabolism and environmental information processing.
**Table S3.** The correlation between relative abundance of orders and soil habitat pH.
**Table S4.** Details of PLS‐PM (Partial Least Squares Path Modeling) for Figure 3A (left).
**Table S5.** Spearman correlation between pedogenic factors, soil habitat properties, microbiome components, and N cycling.
**Table S6.** The information of selected N cycling genes.
**Table S7.** Details of PLS‐PM (Partial Least Squares Path Modeling) for Figure 3A (right).
**Table S8.** Details of PLS‐PM (Partial Least Squares Path Modeling) for Figure 4A.
**Table S9.** Details of PLS‐PM (Partial Least Squares Path Modeling) for Figure 4B.
**Table S10.** Details of PLS‐PM (Partial Least Squares Path Modeling) for Figure 4C.
**Table S11.** Details of PLS‐PM (Partial Least Squares Path Modeling) for Figure 4D.

## Data Availability

The forest site information and the datasets on pedogenic factors, soil habitat properties, and N cycling are available at the Ecological Research Data Paper Archives (https://db.cger.nies.go.jp/JaLTER/ER_DataPapers/archives/2014/ERDP-2014-02/metadata) and https://github.com/kazuo-isobe/GRENE. Sequence data are available in DDBJ databank with the accession number PRJDB18602 for 16S rRNA gene, ITS gene, and shotgun metagenome sequences (https://ddbj.nig.ac.jp/search/entry/bioproject/PRJDB18602). The data and scripts used are saved in GitHub (https://github.com/kazuo-isobe/GRENE). Supplementary materials (methods, figures, tables, graphical abstract, slides, videos, Chinese translated version and update materials) may be found in the online DOI or iMeta Science http://www.imeta.science/imetaomics/.

## References

[1] Rusakova, Elena , Elena Sukhacheva , and Alfred E. Hartemink . 2022. “Vasiliy Dokuchaev – A Biographical Sketch on the Occasion of His 175th Birthday.” Geoderma 412: 115718. 10.1016/j.geoderma.2022.115718

[2] Hall, Ed K. , Emily S. Bernhardt , Raven L. Bier , Mark A. Bradford , Claudia M. Boot , James B. Cotner , Paul A. del Giorgio , et al. 2018. “Understanding How Microbiomes Influence the Systems They Inhabit.” Nature Microbiology 3: 977–982. 10.1038/s41564-018-0201-z 30143799

[3] Isobe, Kazuo , Yuta Ise , Hiroyu Kato , Tomoki Oda , Christian E. Vincenot , Keisuke Koba , Ryunosuke Tateno , Keishi Senoo , and Nobuhito Ohte . 2020. “Consequences of Microbial Diversity in Forest Nitrogen Cycling: Diverse Ammonifiers and Specialized Ammonia Oxidizers.” The ISME Journal 14: 12–25. 10.1038/s41396-019-0500-2 PMC6908637 31481743

[4] Myrold, D. David , and Peter J. Bottomley . 2008. “Nitrogen Mmineralization and Immobilization.” In Nitrogen in Agricultural Systems, edited by J. S. Schepers and W. R. Raun , 157–172. (American Society of Agronomy). 10.2134/agronmonogr49.c5

[5] Schimel, Joshua P. , and Jennifer Bennett . 2004. “Nitrogen Mineralization: Challenges of a Changing Paradigm.” Ecology 85: 591–602. 10.1890/03-8002

[6] Booth, Mary S. , John M. Stark , and Edward Rastetter . 2005. “Controls on Nitrogen Cycling in Terrestrial Ecosystems: a Synthetic Analysis of Literature Data.” Ecological Monographs 75: 139–157. 10.1890/04-0988

[7] Kaye, Jason P. , and Stephen C. Hart . 1997. “Competition for Nitrogen Between Plants and Soil Microorganisms.” Trends in Ecology & Evolution 12: 139–143. 10.1016/S0169-5347(97)01001-X 21238010

[8] Verhagen, Frank J. M. , and Hendrikus J. Laanbroek . 1991. “Competition for Ammonium Between Nitrifying and Heterotrophic Bacteria in Dual Energy‐Limited Chemostats.” Applied and Environmental Microbiology 57: 3255–3263. 10.1128/aem.57.11.3255-3263.1991 PMC183957 16348588

[9] Isobe, Kazuo , Junko Ikutani , Yunting Fang , Muneoki Yoh , Jiangming Mo , Yuichi Suwa , Makoto Yoshida , et al. 2018. “Highly Abundant Acidophilic Ammonia‐Oxidizing Archaea Causes High Rates of Nitrification and Nitrate Leaching in Nitrogen‐Saturated Forest Soils.” Soil Biology and Biochemistry 122: 220–227. 10.1016/j.soilbio.2018.04.021

[10] Schimel, Joshua P . 1995. “Ecosystem Consequences of Microbial Diversity And Community Structure.” In Arctic and Alpine Biodiversity: Patterns, Causes and Ecosystem Consequences, edited by F. Stuart Chapin and Christian Körner , 239–254. (Heidelberg: Springer Berlin). 10.1007/978-3-642-78966-3_17

[11] Schimel, Joshua P. , Jennifer Bennett , and Noah Fierer . 2005. “Microbial Community Composition and Soil Nitrogen Cycling: Is There Really a Connection?” In Biological Diversity and Function in Soils, edited by Richard Bardgett , Michael Usher and David Hopkins , 171–188. (Cambridge, UK: Cambridge University Press). 10.1017/CBO9780511541926.011

[12] Isobe, Kazuo , and Nobuhito Ohte . 2014. “Ecological Perspectives on Microbes Involved in N‐Cycling.” Microbes and Environments 29: 4–16. 10.1264/jsme2.ME13159 PMC4041230 24621510

[13] Wei, Wei , Kazuo Isobe , Yutaka Shiratori , Midori Yano , Sakae Toyoda , Keisuke Koba , Naohiro Yoshida , Haoyang Shen , and Keishi Senoo . 2021. “Revisiting the Involvement of Ammonia Oxidizers and Denitrifiers in Nitrous Oxide Emission from Cropland Soils.” Environmental Pollution 287: 117494. 10.1016/j.envpol.2021.117494 34182387

[14] Rocca, Jennifer D. , Edward K. Hall , Jay T. Lennon , Sarah E. Evans , Mark P. Waldrop , James B. Cotner , Diana R. Nemergut , Emily B. Graham , and Matthew D. Wallenstein . 2015. “Relationships Between Protein‐Encoding Gene Abundance and Corresponding Process Are Commonly Assumed Yet Rarely Observed.” The ISME Journal 9: 1693–1699. 10.1038/ismej.2014.252 PMC4511926 25535936

[15] Trivedi, Pankaj , Manuel Delgado‐Baquerizo , Chanda Trivedi , Hangwei Hu , Ian C. Anderson , Thomas C. Jeffries , Jizhong Zhou , and Brajesh K. Singh . 2016. “Microbial Regulation of the Soil Carbon Cycle: Evidence From Gene–Enzyme Relationships.” The ISME Journal 10: 2593–2604. 10.1038/ismej.2016.65 PMC5113853 27168143

[16] Allison, Steven D. , and Jennifer B. H. Martiny . 2008. “Resistance, Resilience, and Redundancy in Microbial Communities.” Proceedings of the National Academy of Sciences 105: 11512–11519. 10.1073/pnas.0801925105 PMC2556421 18695234

[17] Elrys, Ahmed S. , Ahmad Ali , Huimin Zhang , Yi Cheng , Jinbo Zhang , Zu‐Cong Cai , Christoph Müller , and Scott X. Chang . 2021. “Patterns and Drivers of Global Gross Nitrogen Mineralization in Soils.” Global Change Biology 27: 5950–5962. 10.1111/gcb.15851 34407262

[18] Elrys, Ahmed S. , Zhaoxiong Chen , Jing Wang , Yves Uwiragiye , Ayman M. Helmy , El‐Sayed M. Desoky , Yi Cheng , et al. 2022. “Global Patterns of Soil Gross Immobilization of Ammonium and Nitrate in Terrestrial Ecosystems.” Global Change Biology 28: 4472–4488. 10.1111/gcb.16202 35445472

[19] Li, Zhaolei , Dashuan Tian , Bingxue Wang , Jinsong Wang , Song Wang , Han Y. H. Chen , Xiaofeng Xu , et al. 2019. “Microbes Drive Global Soil Nitrogen Mineralization and Availability.” Global Change Biology 25: 1078–1088. 10.1111/gcb.14557 30589163

[20] Malik, Ashish A. , Jeremy Puissant , Kate M. Buckeridge , Tim Goodall , Nico Jehmlich , Somak Chowdhury , Hyun Soon Gweon , et al. 2018. “Land Use Driven Change in Soil pH Affects Microbial Carbon Cycling Processes.” Nature Communications 9: 3591. 10.1038/s41467-018-05980-1 PMC6123395 30181597

[21] Malik, Ashish A. , Jeremy Puissant , Tim Goodall , Steven D. Allison , and Robert I. Griffiths . 2019. “Soil Microbial Communities With Greater Investment in Resource Acquisition Have Lower Growth Yield.” Soil Biology and Biochemistry 132: 36–39. 10.1016/j.soilbio.2019.01.025

[22] Malik, Ashish A. , Jennifer B. H. Martiny , Eoin L. Brodie , Adam C. Martiny , Kathleen K. Treseder , and Steven D. Allison . 2020. “Defining Trait‐Based Microbial Strategies With Consequences for Soil Carbon Cycling Under Climate Change.” The ISME Journal 14: 1–9. 10.1038/s41396-019-0510-0 PMC6908601 31554911

[23] Philippot, Laurent , Aymé Spor , Catherine Hénault , David Bru , Florian Bizouard , Christopher M. Jones , Amadou Sarr , and Pierre‐Alain Maron . 2013. “Loss in Microbial Diversity Affects Nitrogen Cycling in Soil.” The ISME Journal 7: 1609–1619. 10.1038/ismej.2013.34 PMC3721106 23466702

[24] Trivedi, Chanda , Manuel Delgado‐Baquerizo , Kelly Hamonts , Kaitao Lai , Peter B. Reich , and Brajesh K. Singh . 2019. “Losses in Microbial Functional Diversity Reduce the Rate of Key Soil Processes.” Soil Biology and Biochemistry 135: 267–274. 10.1016/j.soilbio.2019.05.008

[25] Delgado‐Baquerizo, Manuel , Peter B. Reich , Chanda Trivedi , David J. Eldridge , Sebastián Abades , Fernando D. Alfaro , Felipe Bastida , et al. 2020. “Multiple Elements of Soil Biodiversity Drive Ecosystem Functions Across Biomes.” Nature Ecology & Evolution 4: 210–220. 10.1038/s41559-019-1084-y 32015427

[26] Norton, Jeanette , and Yang Ouyang . 2019. “Controls and Adaptive Management of Nitrification in Agricultural Soils.” Frontiers in Microbiology 10: 1931. 10.3389/fmicb.2019.01931 PMC6728921 31543867

[27] Aber, John D. , Christine L. Goodale , Scott V. Ollinger , Marie‐Louise. Smith , Alison H. Magill , Mary E. Martin , Richard A. Hallett , and John L. Stoddard . 2003. “Is Nitrogen Deposition Altering the Nitrogen Status of Northeastern Forests?” BioScience 53: 375–389. 10.1641/0006-3568(2003)053[0375:INDATN]2.0.CO;2

[28] Li, Zhaolei , Zhaoqi Zeng , Dashuan Tian , Jinsong Wang , Zheng Fu , Fangyue Zhang , Ruiyang Zhang , et al. 2020. “Global Patterns and Controlling Factors of Soil Nitrification Rate.” Global Change Biology 26: 4147–4157. 10.1111/gcb.15119 32301539

[29] Huber, Katharina J. , and Jörg Overmann . 2018. “ *Vicinamibacteraceae* fam. nov., the First Described Family Within the Subdivision 6 Acidobacteria.” International Journal of Systematic and Evolutionary Microbiology 68: 2331–2334. 10.1099/ijsem.0.002841 29809123

[30] Wang, Wenbo , Yuanyuan Yang , Jinge Li , Pengtu Bu , Aijun Lu , Hao Wang , Wenxing He , Ramon Santos Bermudez , and Jian Feng . 2024. “Consecutive Fertilization‐Promoted Soil Nutrient Availability and Altered Rhizosphere Bacterial and Bulk Fungal Community Composition.” Forests 15: 514. 10.3390/f15030514

[31] Qi, Daihua , Xuwen Wieneke , Peipei Xue , Li He , and Udaya DeSilva . 2021. “Total Nitrogen Is the Main Soil Property Associated With Soil Fungal Community in Karst Rocky Desertification Regions in Southwest China.” Scientific Reports 11: 10809. 10.1038/s41598-021-89448-1 PMC8144601 34031439

[32] Telagathoti, Anusha , Maraike Probst , and Ursula Peintner . 2021. “Habitat, Snow‐Cover and Soil pH, Affect the Distribution and Diversity of Mortierellaceae Species and Their Associations to Bacteria.” Frontiers in Microbiology 12: 669784. 10.3389/fmicb.2021.669784 PMC8283828 34276602

[33] Owaresat, J. K. , M. A. Habib Siam , D. Dey , S. Jabed , F. Badsha , M. R. Islam , and M. S. Kabir . 2023. “Factors Impacting Rhizobium‐Legume Symbiotic Nitrogen Fixation with the Physiological and Genetic Responses to Overcome the Adverse Conditions: A Review.” Agricultural Reviews 44: 22–30. 10.18805/ag.RF-257

[34] Zhang, Tao , Neng‐Fei Wang , Hong‐Yu Liu , Yu‐Qin Zhang , and Li‐Yan Yu . 2016. “Soil pH Is a Key Determinant of Soil Fungal Community Composition in the Ny‐Ålesund Region, Svalbard (High Arctic).” Frontiers in Microbiology 7: 227. 10.3389/fmicb.2016.00227 PMC4767930 26955371

[35] Fernandez, Christopher W. , and Craig R. See . 2025. “The pH Influence on Ectomycorrhizal Nitrogen Acquisition and Decomposition.” New Phytologist 246: 867–875. 10.1111/nph.70021 PMC11982800 40065484

[36] Kalam, Sadaf , Anirban Basu , Iqbal Ahmad , R. Z. Sayyed , Hesham Ali El‐Enshasy , Daniel Joe Dailin , and Ni Luh Suriani . 2020. “Recent Understanding of Soil Acidobacteria and Their Ecological Significance: A Critical Review.” Frontiers in Microbiology 11: 580024. 10.3389/fmicb.2020.580024 PMC7661733 33193209

[37] Kielak, Anna M. , Cristine C. Barreto , George A. Kowalchuk , Johannes A. van Veen , and Eiko E. Kuramae . 2016. “The Ecology of Acidobacteria: Moving Beyond Genes and Genomes.” Frontiers in Microbiology 7: 744. 10.3389/fmicb.2016.00744 PMC4885859 27303369

[38] Bruyant, Pauline , Yvan Moënne‐Loccoz , and Juliana Almario . 2024. “Root‐Associated Helotiales Fungi: Overlooked Players in Plant Nutrition.” Soil Biology and Biochemistry 191: 109363. 10.1016/j.soilbio.2024.109363

[39] Suelgaray, Fernando Javier Ureta , Viviana Mónica Chiocchio , Federico Ciolfi , and Mario Carlos Nazareno Saparrat . 2023. “Are Dark Septate Endophytes an Ancestral Ecological State in the Evolutionary History of the Order Chaetothyriales?” Archives of Microbiology 205: 55. 10.1007/s00203-023-03401-6 36607426

[40] Graham, Emily B. , Joseph E. Knelman , Andreas Schindlbacher , Steven Siciliano , Marc Breulmann , Anthony Yannarell , J. M. Beman , et al. 2016. “Microbes as Engines of Ecosystem Function: When Does Community Structure Enhance Predictions of Ecosystem Processes?” Frontiers in Microbiology 7: 214. 10.3389/fmicb.2016.00214 PMC4764795 26941732

[41] Fierer, Noah , Jonathan W. Leff , Byron J. Adams , Uffe N. Nielsen , Scott Thomas Bates , Christian L. Lauber , Sarah Owens , et al. 2012. “Cross‐Biome Metagenomic Analyses of Soil Microbial Communities and Their Functional Attributes.” Proceedings of the National Academy of Sciences 109: 21390–21395. 10.1073/pnas.1215210110 PMC3535587 23236140

[42] Luan, Lu , Yuji Jiang , Francisco Dini‐Andreote , Thomas W. Crowther , Pengfa Li , Mohammad Bahram , Jie Zheng , et al. 2023. “Integrating pH Into the Metabolic Theory of Ecology to Predict Bacterial Diversity in Soil.” Proceedings of the National Academy of Sciences 120: e2207832120. 10.1073/pnas.2207832120 PMC9934207 36626561

[43] Kobayashi, Hiroshi , Hiromi Saito , and Tomohito Kakegawa . 2000. “Bacterial Strategies to Inhabit Acidic Environments.” The Journal of General and Applied Microbiology 46: 235–243. 10.2323/jgam.46.235 12483574

[44] Piton, Gabin , Steven D. Allison , Mohammad Bahram , Falk Hildebrand , Jennifer B. H. Martiny , Kathleen K. Treseder , and Adam C. Martiny . 2023. “Life History Strategies of Soil Bacterial Communities Across Global Terrestrial Biomes.” Nature Microbiology 8: 2093–2102. 10.1038/s41564-023-01465-0 37798477

[45] Fierer, Noah . 2017. “Embracing the Unknown: Disentangling the Complexities of the Soil Microbiome.” Nature Reviews Microbiology 15: 579–590. 10.1038/nrmicro.2017.87 28824177

[46] Isobe, Kazuo , Keisuke Koba , Shigeto Otsuka , and Keishi Senoo . 2011. “Nitrification and Nitrifying Microbial Communities in Forest Soils.” Journal of Forest Research 16: 351–362. 10.1007/s10310-011-0266-5

[47] Stark, John M. , and Stephen C. Hart . 1997. “High Rates of Nitrification and Nitrate Turnover in Undisturbed Coniferous Forests.” Nature 385: 61–64. 10.1038/385061a0

[48] Fang, Yunting , Muneoki Yoh , Keisuke Koba , Weixing Zhu , Y. U. Takebayashi , Yihua Xiao , Chunyi Lei , et al. 2011. “Nitrogen Deposition and Forest Nitrogen Cycling Along an Urban–Rural Transect in Southern China.” Global Change Biology 17: 872–885. 10.1111/j.1365-2486.2010.02283.x

[49] Aber, John , William McDowell , Knute Nadelhoffer , Alison Magill , Glenn Berntson , Mark Kamakea , Steven McNulty , et al. 1998. “Nitrogen Saturation in Temperate Forest Ecosystems.” BioScience 48: 921–934. 10.2307/1313296

[50] Malik Ashish, A. , C. Thomson Bruce , S. Whiteley Andrew , Mark Bailey , and I. Griffiths Robert . 2017. “Bacterial Physiological Adaptations to Contrasting Edaphic Conditions Identified Using Landscape Scale Metagenomics.” Mbio 8: e00799‐17. 10.1128/mbio.00799-17 PMC5573673 28679747

[51] Wang, Cong , Qing‐Yi Yu , Niu‐Niu Ji , Yong Zheng , John W. Taylor , Liang‐Dong Guo , and Cheng Gao . 2023. “Bacterial Genome Size and Gene Functional Diversity Negatively Correlate With Taxonomic Diversity Along a pH Gradient.” Nature Communications 14: 7437. 10.1038/s41467-023-43297-w PMC10656551 37978289

[52] Urakawa, Rieko , Nobuhito Ohte , Hideaki Shibata , Kazuo Isobe , Ryunosuke Tateno , Tomoki Oda , Takuo Hishi , et al. 2016. “Factors Contributing to Soil Nitrogen Mineralization and Nitrification Rates of Forest Soils in the Japanese Archipelago.” Forest Ecology and Management 361: 382–396. 10.1016/j.foreco.2015.11.033

[53] Hart, Stephen C. , John M. Stark , Eric A. Davidson , and Mary K. Firestone . 1994. “Nitrogen Mineralization, Immobilization, and Nitrification.” In Methods of Soil Analysis: Part 2 Microbiological and Biochemical Properties, edited by R. W. Weaver , Scott Angle , Peter Bottomley , David Bezdicek , Scott Smith , Ali Tabatabai and Art Wollum , 985–1018. (Cambridge, UK: Soil Science Society of America, Inc.). 10.2136/sssabookser5.2.c42

[54] Urakawa, Rieko , Nobuhito Ohte , Hideaki Shibata , Ryunosuke Tateno , Takuo Hishi , Keitaro Fukushima , Yoshiyuki Inagaki , et al. 2015. “Biogeochemical Nitrogen Properties of Forest Soils in the Japanese Archipelago.” Ecological Research 30: 1–2. 10.1007/s11284-014-1212-8

[55] Kuroiwa, Megumi , Keisuke Koba , Kazuo Isobe , Ryunosuke Tateno , Asami Nakanishi , Yoshiyuki Inagaki , Hiroto Toda , et al. 2011. “Gross Nitrification Rates in Four Japanese Forest Soils: Heterotrophic Versus Autotrophic and the Regulation Factors for the Nitrification.” Journal of Forest Research 16: 363–373. 10.1007/s10310-011-0287-0

[56] Edgar, Robert C . 2013. “UPARSE: Highly Accurate OTU Sequences from Microbial Amplicon Reads.” Nature Methods 10: 996–998. 10.1038/nmeth.2604 23955772

[57] Rognes, Torbjørn , Tomáš Flouri , Ben Nichols , Christopher Quince , and Frédéric Mahé . 2016. “VSEARCH: A Versatile Open Source Tool for Metagenomics.” PeerJ 4: e2584. 10.7717/peerj.2584 PMC5075697 27781170

[58] Estaki, Mehrbod , Lingjing Jiang , Nicholas A. Bokulich , Daniel McDonald , Antonio González , Tomasz Kosciolek , Cameron Martino , et al. 2020. “QIIME 2 Enables Comprehensive End‐to‐End Analysis of Diverse Microbiome Data and Comparative Studies With Publicly Available Data.” Current Protocols in Bioinformatics 70: e100. 10.1002/cpbi.100 PMC9285460 32343490

[59] Takami, Hideto . 2024. “Functional Microbial Diversity: Functional Genomics and Metagenomics Using Genomaple.” In Microbial Diversity in the Genomic Era: Functional Diversity and Community Analysis, edited by Surajit Das and Hirak Ranjan Dash , 439–465. (California, USA: Academic Press). 10.1016/B978-0-443-13320-6.00026-3

[60] Takami, Hideto , Takeaki Taniguchi , Wataru Arai , Kazuhiro Takemoto , Yuki Moriya , and Susumu Goto . 2016. “An Automated Systemfor Evaluation of the Potential Functionome: MAPLE Version 2.1.0.” DNA Research 23: 467–475. 10.1093/dnares/dsw030 PMC5066172 27374611

[61] R Core Team . 2020. “R: A Language and Environment for Statistical Computing.” R Foundation for Statistical Computing, Vienna, Austria. https://www.R-project.org/

[62] Ferrier, Simon , Glenn Manion , Jane Elith , and Karen Richardson . 2007. “Using Generalized Dissimilarity Modelling to Analyse and Predict Patterns of Beta Diversity in Regional Biodiversity Assessment.” Diversity and Distributions 13: 252–264. 10.1111/j.1472-4642.2007.00341.x

[63] Sanchez, Gaston , Giorgio Russolillo , and Laura Trinchera . 2015. plspm: Tools for Partial Least Squares Path Modeling (PLS‐PM). 10.32614/CRAN.package.plspm

[64] Wetzels, Martin , Gaby Odekerken‐Schröder , and O. van Oppen . 2009. “Using PLS Path Modeling for Assessing Hierarchical Construct Models: Guidelines and Empirical Illustration.” MIS Quarterly 33: 177–195. 10.2307/20650284

