# Supplementary material for: Soil pH modulates microbial nitrogen allocation in soil via compositional and metabolic shifts across forests in Japan
Source: IMetaOmics. 2025 Sep 18;2(4):e70054. doi: 10.1002/imo2.70054 (PMC12805986; doi:10.1002/imo2.70054)
Supplement: Supplementary file 1 — Figure S1. Taxonomic composition shifts in response to soil habitat pH. Figure S2. Shifts in taxonomic composition of bacterial and fungal communities at the order level in response to soil pH. Figure S3. Shifts in taxonomic and metabolic composition in response to soil pH. Figure S4. Spearman correlations among KEGG modules that were significantly associated with soil pH. [file IMO2-2-e70054-s001.docx]

Supporting information to

**Soil pH modulates microbial nitrogen allocation in soil via compositional and metabolic shifts across forests in Japan**

**Running title:** Microbial community response and N-cycling outcome

Yaping Liu^1,2#^, Yuta Ise^3,4#^, Hideto Takami^5,6^, Rieko Urakawa^7^, Ryunosuke Tateno^8^, Atsushi Toyoda^9^, Nobuhito Ohte^10^, Weiyu Shi^11^, Lin Jiang^12^, Kazuo Isobe^1,2^*

^1^Institute of Ecology, College of Urban and Environmental Sciences, Peking University, Beijing, 100080, China

^2^State Key Laboratory of Vegetation Structure, Function and Construction (VegLab), Peking University, Beijing, 100080, China

^3^Graduate School of Agricultural and Life Sciences, The University of Tokyo, Tokyo, 113-8657, Japan

^4^Institute for Agro-Environmental Sciences, National Agriculture and Food Research Organization, Ibaraki, 305-8604, Japan

^5^Faculty of Agriculture, Tokyo University of Agriculture and Technology, Tokyo, 183-8509, Japan

^6^Center for Mathematical Science and Advanced Technology, JAMSTEC, Yokohama, 236-0001, Japan

^7^Asia Center for Air Pollution Research, Japan Environmental Sanitation Center, Niigata, 950-2144, Japan

^8^Field Science Education and Research Center, Kyoto University, Kyoto, 606-8502, Japan

^9^Comparative Genomics Laboratory, National Institute of Genetics, Shizuoka, 411-8540, Japan

^10^Graduate School of Informatics, Kyoto University, Kyoto, 606-8501, Japan

^11^School of Geographical Sciences, Southwest University, Chongqing, 400715, China

^12^School of Biological Sciences, Georgia Institute of Technology, Georgia, 30332, USA

^#^ These authors contributed equally: Yaping Liu, Yuta Ise

*Correspondence: kazuo.isobe@gmail.com (Kazuo Isobe)

**METHODS**

**Study sites and sampling**

The datasets used in this study covered 40 forested sites across Japan (Figure 1A). The latitude ranged from 44°22' N to 26°45' N. The longitude ranged from 144°39' E to 128°13' E. Mean annual temperature (MAT) ranged from 4.4 to 20.9 °C, and mean annual precipitation (MAP) ranged from 820 to 3080 mm. The climatic zones for most sites were temperate, with one site being subtropical. Among the study sites, there were 15 broadleaved forests consisting of Japanese oak (*Quercus crispula*), Japanese beech (*Fagus crenata*), Konara oak (*Quercus serrata*), *Ilex pedunculosa*, *Castanopsis sieboldii*, *Quercus acutissima*, and *Tilia maximowicziana*. The other 25 sites were coniferous forests of Japanese cedar (*Cryptomeria japonica*), cypress (*Chamaecyparis obtusa*), larch (*Larix kaempferi*), red pine (*Pinus densiflora*), *Abies sachalinensis*, and *Abies firma*. The soil types were classified based on the IUSS Working Group World Reference Base (2014) into Cambisols (17 sites), Andosols (18 sites), Regosols (4 sites), and Acrisols (1 site) (Table S1).

To create the map of pedogenic factors (*i.e.*, MAT, MAP, vegetation, and soil types) across Japan, we downloaded the shapefile data from the Ministry of Land, Infrastructure, Transport, and Tourism (https://nlftp.mlit.go.jp/ksj/gml/datalist/KsjTmplt-G02-v3_0.html) for MAT and MAP, the GeoTIFF file from the Earth Observation Research Center (EORC) of the Japan Aerospace Exploration Agency (JAXA) (https://www.eorc.jaxa.jp/ALOS/en/dataset/lulc_e.htm) for vegetation, and the shapefile from the Institute for Agro-Environmental Sciences of National Agriculture and Food Research Organization (NARO) (https://soil-inventory.rad.naro.go.jp) for soil types. The map was created using the “ggplot2” package in R (v4.3.2).

We conducted a sampling of mineral soil layers once at each forest site, either in summer or autumn, avoiding periods of extreme soil moisture conditions, such as the rainy season (early summer). We collected mineral soils from 0 to 10 cm depth at five locations at each forest site as described previously [1].

**Soil chemical analyses and gross N transformation rate measurement**

For soil habitat properties, we measured the C and N contents of mineral soils using a CN analyzer after air-drying and grinding the samples. We also measured soil pH (H_2_O) with a glass electrode after water extraction (fresh soil to deionized water ratio of 1:1.25, shaken for 1 h).

For N cycling rate and allocation, we measured gross N transformation rates in soil using the ^15^N isotope dilution method [2]. The measurements followed previously described methods [3,4]. Briefly, 7 grams of fresh soil, supplemented with 1 mL of 1 mM ^15^NH_4_Cl (99.7% ^15^N atom) or Na^15^NO_3_ (99.8% ^15^N atom), were incubated at 20 °C. NH_4_^+^ and NO_3_^−^ were extracted from the soils with 35 mL of 2 M-KCl solution after 2 and 26 h of incubation by shaking for 1 h. The concentrations of NH_4_^+^ and NO_3_^−^ in the extracts were measured colorimetrically. NH_4_^+^ was trapped on acidified filter paper disks, followed by its persulfate oxidation to NO_3_^−^ [5]. The denitrifier method was then used for measuring the ^15^N atom% of NH_4_^+^ and NO_3_^−^ using gas chromatography with a mass spectrometer (GCMS-QP2010 Plus, Shimadzu Corp., Kyoto, Japan) after conversion of NO_3_^−^-N into N_2_O [5]. Gross ammonification, ammonium consumption, nitrification, and nitrate consumption rates were calculated according to the methods described by Hart SC et al [2]. The gross ammonium immobilization or uptake rate was calculated as ammonium consumption minus nitrification [6].

We have previously reported the soil chemical properties and gross N transformation rates for most forest, making the data available sites [1,3]. We obtained new data for five additional sites in this study.

**rRNA gene quantification and sequencing**

We analyzed the abundance of bacterial and fungal communities by rRNA gene quantification by quantitative polymerase chain reaction (qPCR), using 200 samples with 5 replicates from 40 forests. Microbial community DNA was extracted from 0.4 g of mineral soil and purified using an ISOIL kit for bead beating (Nippon Gene, Tokyo, Japan). For bacteria, we targeted the V4 region of the 16S rRNA gene using the primer pair 515F/806R [7]. For fungi, we targeted the V1–V2 region of the 18S rRNA gene using the primer pair GC-Fung/NS1 [8]. Each reaction was performed in a 20–25 μL volume, using 1 × SYBR Premix Ex Taq II (Takara Bio, Shiga, Japan). The forward and reverse primers were used at 0.2 μM each, and 10 ng of DNA was used as the template. The reactions were conducted using a StepOnePlus system (Applied Biosystems, Foster City, CA). The PCR conditions for bacterial amplification were as follows: an initial denaturation at 95 °C for 30 s, followed by 40 cycles of 95 °C for 5 s, 55 °C for 30 s, and 72 °C for 30 s. For fungal amplification, the PCR conditions were: an initial denaturation at 95 °C for 5 s, followed by 40 cycles of 95 °C for 5 s, 50 °C for 30 s, and 72 °C for 30 s. To generate standard curves for quantification, genomic DNA from pure bacterial strains (*Pseudomonas stutzeri* JCM 5965) and fungal strains (*Fusarium oxysporum* COF-2) [9] were used as templates. The target genes were cloned and plasmids were extracted using the QIAGEN Plasmid Mini Kit (Qiagen, Valencia, CA). The plasmids were linearized using the restriction enzyme ScaI (Nippon Gene) and serially diluted to generate standard curves. The amplification efficiencies of all genes used for standard curve generation were > 90%, and the standard curves had high correlation coefficients (R^2^ > 0.95).

We analyzed the taxonomic composition and richness by 16S rRNA and ITS gene sequencing for bacterial and fungal communities, respectively (200 samples with 5 replicates from 40 forest). The prokaryotic 16S rRNA genes were sequenced and analyzed to estimate the richness and composition of bacterial communities. The PCR primers (515F/806R) contained the appropriate Illumina adapters and 12-bp barcodes for multiplex sequencing on the Illumina platform [10]. The PCR mixture (30 μL) contained 1× Ex Taq buffer (Takara Bio, Shiga, Japan), 0.2 mM of each dNTP, 0.2 μM of each primer, 2 U/100 μL of TaKaRa Ex Taq HS (Takara Bio), and 10 ng of DNA template. The amplification was performed under the following conditions: initial denaturation at 94 °C for 3 min, followed by 21 cycles of 94 °C for 30 s, 50 °C for 45 s, and 72 °C for 90 s, with a final extension at 72 °C for 5 min. The PCR amplicons were purified by gel excision after electrophoresis using the Wizard SV Gel and PCR Clean-Up System (Promega, WI, USA), quantified using a Qubit dsDNA HS kit (Life Technologies Inc., CA, USA), and pooled at equimolar concentrations. The libraries were quality checked for concentration and amplicon size using an Agilent 2100 Bioanalyzer (Agilent Technologies, CA, USA) and sequenced using a MiSeq Reagent Kit V2 500 Cycle (Illumina, Inc., CA, USA) on an Illumina MiSeq platform.

The fungal ITS genes (ITS2 region) were sequenced and analyzed to estimate the richness and composition of fungal communities. A two-step PCR method was used to prepare samples for sequencing. First, soil DNA was used as a template for PCR to amplify the ITS2 region of all fungi using the primers ITS3ngsmix1–5 / ITS4ngs [11] (1st PCR). The PCR mixture (20 µL) consisted of 1× PCR Buffer for KOD-Plus-Neo (Toyobo, Osaka, Japan), 1.0 mM MgSO_4_, 0.2 mM of each dNTP, 0.3 µM of each forward and reverse primer, 1 U/50 µL of KOD-Plus-Neo (Toyobo), and 10 ng of DNA sample. The PCR conditions were set as follows: an initial step at 94 °C for 3 min, followed by 35 cycles of 94 °C for 30 s, 50 °C for 45 s, and 68 °C for 1 min, with a final step at 68 °C for 5 min. Next, a second PCR (2nd PCR) was performed using the 1st PCR products as templates to add adapter and index sequences. The reaction mixture for each sample (30 µL) consisted of 1× PCR Buffer for KOD-Plus-Neo, 0.8 mM MgSO_4_, 0.2 mM of each dNTP, 0.3 µM of each forward and reverse primer, 1 U/50 µL of KOD-Plus-Neo, and 1.5 µL of 1st PCR product. The PCR conditions were set as follows: an initial step at 94 °C for 3 min, followed by 8 cycles of 94 °C for 30 s, 50 °C for 45 s, and 68 °C for 1 min, with a final step at 68 °C for 5 min. The primers (P5-adaptor / P7-adaptor) were designed to anneal to the sequencing primer binding sites of the 1st PCR products and included adapter sequences necessary for MiSeq sequencing. The reverse primer (P7-adaptor) also included a 12 bp barcode sequence to differentiate between samples. The 2nd PCR products were purified as described above. The DNA from each sample was then combined in equal amounts to prepare the sample library, which was sequenced using a MiSeq Reagent Kit V3 600 Cycles (Illumina).

The 16S rRNA gene and ITS2 region sequencing data obtained from MiSeq were processed using UPARSE [12], VSEARCH (v2.22.1) [13], and QIIME2 (v2023.2) [14]. First, paired-end reads with at least 30 bases overlapping from both forward and reverse reads were merged, and sequences with more than 0.5 bases of low-quality scores were excluded. Representative sequences were then extracted from the “full sequences” of the target genes, singletons were removed, and OTU clustering was performed to identify the amplicon sequence variant (ASV). Chimeric sequences were excluded by referring to the RDP Gold database (http://drive5.com/uchime/rdp_gold_fa) for 16S rRNA genes. For the ITS2 region, the VSEARCH uchime3_denovo algorithm was used to detect chimeric sequences. The taxonomy of each ASV was assigned using the sklearn classifier, referencing the SILVA database (v138.1) [15] for the 16S rRNA gene and UNITE database (v9.0) [16] for ITS2 region. Because a variable number of sequence-reads per sample (22,055–137,962 reads for bacteria and 90,138–317,259 reads for fungi) was obtained, the sequence data were rarefied to the lowest number of reads (22,055 reads for bacteria and 90,138 reads for fungi). The richness (the number of ASVs) of bacterial and fungal communities was calculated using the “phyloseq” package [17].

**Shotgun metagenomic sequencing and analysis**

We analyzed the metabolic capabilities of microbial communities using shotgun metagenomic sequencing. In this study, we characterized microbial metabolic capabilities by using the composition and abundance of the Kyoto Encyclopedia of Genes and Genomes (KEGG) functional modules, which represent the community-level potential to perform specific metabolic functions. Module abundance was used as a quantitative proxy for these capabilities and was normalized across samples to allow for comparison.

For metagenomic analysis, one replicate from each site was selected for sequencing (40 samples, 1 sample from each forest). DNA samples extracted from soil were prepared to have a DNA concentration of at least 100 ng/µL and a yield of at least 5 µg. If the DNA concentration or yield was insufficient, additional DNA extraction was performed from the same soil sample. Shotgun libraries were prepared using the HiSeq Rapid SBS kit v2 (insert size: 450 base pairs; Illumina, San Diego, CA) with the prepared DNA samples. The libraries were sequenced on a HiSeq 2500 (Illumina) with 251 bp paired-end reads (251 bp sequencing from both forward and reverse). Base calling of the sequences was performed using RTA 1.18.64 (Illumina). Raw paired-end reads were first quality-filtered to retain only those with a minimum Phred score of Q20. The sequences were merged if they had an overlap of at least 10 bases using PEAR [18]. Merged reads shorter than 400 bp were excluded. ORFs were predicted using MetaGeneAnnotator [19], translated into amino acid sequences using EMBOSS [20], and filtered to retain sequences ≥ 100 amino acids. A total of 3 million amino acid sequences were randomly subsampled per sample for downstream analysis.

The translated amino acid sequences were analyzed using Genomaple (Genome Metabolic and Physiological Potential Evaluator) [21−23], which is designed to evaluate microbial metabolic potential by assigning KEGG Orthology (KO) identifiers and mapping them to functional modules defined in the Kyoto Encyclopedia of Genes and Genomes (KEGG). KO assignment was performed using the KEGG Automatic Annotation Server (KAAS), with homology searches conducted via the single-directional best hit (SBH) algorithm implemented in GHOSTX. The KO-assigned sequences were mapped to 814 KEGG functional modules, including pathways, complexes, functional sets, and signatures. Genomaple is available as a stand-alone package from Docker Hub (https://hub.docker.com/r/genomaple/genomaple), enabling reproducible analysis of metagenomic datasets. The abundance of each KO was calculated by dividing the total number of reads assigned to that KO by its average gene length. For each module, abundance was determined by mapping all KOs involved in its reaction steps and taking the minimum value among the normalized KO abundances across all steps. To enable cross-sample comparison, module abundance was further normalized using the abundance of a virtual ribosomal module (M90000), which consists of 79 ribosomal proteins (31 KOs) conserved across bacteria and archaea, and serves as a proxy for total microbial cell abundance.

The taxonomic composition based on metagenomic data was analyzed using the total KO mapped to the virtual ribosome module (M91000) [21]. Since the number of ribosomal proteins differs by domain (52 for bacteria, 58 for archaea, and 77 for eukaryotes), the total KO for each domain was used directly for bacteria, and the totals for archaea and eukaryotes were adjusted by multiplying by 52/58 and 52/77, respectively, to allow for domain-level comparisons.

Genes assigned to the KO involved in the modules of the “Nitrogen metabolism” pathway (M00175, M00531, M00530, M00529, M00528, M00804, and M00973) were utilized as N-cycling KO genes. Then the richness of N-cycling genes was calculated using number of KO genes.

**Statistical analyses**

All statistical analyses were performed using R (v4.3.2) [24]. We conducted classification random forest analyses to explore the relative importance of pedogenic factors in shaping soil habitat properties. In our models, different pedogenic factors (MAP, MAT, vegetation, and soil type) were included as predictors, and soil habitat properties (soil pH, and soil C and N contents) were the response variables. Next, we conducted random forest analyses to explore the relative importance of pedogenic factors and soil habitat properties in shaping the abundance and richness of bacterial and fungal communities. These analyses were conducted using the “rfPermute” package [25]. Additionally, we performed a linear correlation analysis between the most significant predictor and the response variables. Differences in soil pH among vegetations and in soil C and N among soil types were evaluated using the Kruskal–Wallis test.

We conducted generalized dissimilarity modeling (GDM) [26] to explore the relative contributions of pedogenic factors and soil habitat properties to taxonomic compositions. GDM is a statistical modeling method that assumes nonlinear relationships between predictors and response variables, expressed as distance matrices. It is particularly suited for microbial ecological data, as it models nonlinear associations between environmental variables and beta-diversity using dissimilarity matrices. We used ASV data for bacteria and fungi and calculated the dissimilarity of composition using the Bray–Curtis index as a distance matrix with the “phyloseq” package. The predictors included MAT, MAP, vegetation, soil type, soil pH, soil C and N contents, soil C-to-N ratio, and geographical distance between sites. Euclidean distance was calculated for these factors (note that vegetation and soil type were converted into binary data prior to distance matrix calculation). GDM was performed using the “gdm” package in R (v1.3.4) [27]. All data used as predictors were standardized using the “BBmisc” package [28] to allow for direct comparison of relative contributions. Then, backward elimination was used for predictor selection in modeling. This process involved initial modeling with all predictors, followed by stepwise removal of the variable with the highest *p* value among those deemed statistically insignificant (*p* > 0.05). Modeling was repeated with the remaining variables until all variables were significant (*p* < 0.05), with 100 permutations for each step. GDM was conducted using the selected predictors, and I-spline curves and deviance explained were obtained for each predictor. We further conducted non-metric multidimensional scaling (NMDS) using the Bray–Curtis distance matrix to explore the similarity of bacterial and fungal taxonomic compositions between samples with the “phyloseq” package. Because GDM analysis identified soil pH as the most significant predictor for both bacterial and fungal community compositions, we used permutational multivariate ANOVA (PERMANOVA) with 1000 permutations to determine if soil pH explains the variation in composition across samples. Additionally, we performed a linear correlation analysis between soil pH and the NMDS1 scores.

Using the abundance data of all functional modules and the phylum-level taxonomic composition data based on the module of ribosomal protein genes (M91000), we calculated the dissimilarity of functional module composition and taxonomic composition using the Bray–Curtis distance. Based on this distance matrix, we conducted NMDS to evaluate the similarity of the compositions. We used PERMANOVA to determine if soil pH explains the variation in compositions across samples. Additionally, we performed a linear correlation analysis between NMDS1 scores of taxonomic composition and NMDS2 scores of functional module composition. We also performed a correlation analysis by calculating Spearman’s rank correlation coefficient between the abundance of each functional module and soil pH.

**Structural equation modeling**

We used the partial least squares path modeling (PLS-PM) [29] to examine the hierarchical effects from pedogenic factors, soil habitat properties, and soil microbial community components to N cycling. We employed partial least squares path modeling (PLS-PM). PLS-PM is well-suited for our dataset because it (i) accommodates multicollinearity among variables (*e.g.*, soil carbon and nitrogen contents), (ii) does not require multivariate normality, and (iii) is effective with small to moderate sample sizes. Additionally, PLS-PM allows for the construction of latent variables to represent complex ecological constructs, such as “nitrogen cycling rate”. However, it should be noted that, while conceptually powerful and flexible, PLS-PM is a variance-based SEM method and may be less statistically rigorous than covariance-based SEM in terms of model fit and inference. We used both manifest variables and latent variables to test our hypothesis. During PLS-PM implementation, we excluded two sites (TMC and TMN) due to the missing data on N cycling rates. Consequently, we used data from 38 sites. For PLS-PM on the abundance and taxonomic composition, we used *n* = 190 (five replicates from each site), and for PLS-PM on taxonomic richness, we used *n* = 38 (one replicate from each site) because we asked if the greater richness leads to the greater richness of N cycling genes. Vegetation data and soil type data were numerically categorized, and other data were standardized as continuous variables. For taxonomic composition, we used NMDS1 scores for bacteria and fungi to represent taxonomic composition. For samples where gross ammonification and nitrification rates were below detection limits, we treated the measurements as 0 mg N kg^−1^ d^−1^ to avoid missing values. N cycling rate was conceptualized as a latent variable reflecting gross rates of ammonification, nitrification, and ammonium or nitrate consumption, where higher values indicated increased rates. N allocation was also defined as a latent variable, comprising ratios of nitrification-to-ammonification and the inverse of ammonium immobilization (as uptake)-to-ammonification (as ammonium release), with higher values denoting a more open N cycling prone to nitrate loss. N cycling gene richness was represented by the number of N-cycling genes in metagenomes.

We conducted PLS-PM by using the R package “plspm” [29]. The path coefficients were estimated using the centroid weighting scheme based on standardized data. These coefficients represent the strength and direction of the relationships between the latent variables. The significance of path coefficients was assessed using bootstrapping with 1000 resamples, and *p* < 0.05 were considered statistically significant. The coefficient of determination, R², for endogenous latent variables represents the proportion of variance explained by their predictors. Higher R² values indicate stronger explanatory power of the model for that latent variable. The goodness of fit index is used to evaluate the overall quality of the PLS-PM model. It is calculated as the square root of the product of the average R² of endogenous latent variables and the average AVE (Average Variance Extracted) of all latent variables. In our PLS-PM, the goodness of fit value ranged from 0.40 to 0.60, which exceeds the commonly accepted threshold of 0.36 for large effect sizes [30], indicating a satisfactory global model fit. The evaluation results of the mode are summarized in Tables S6−11.

**REFERENCES**

1. Urakawa, Rieko, Nobuhito Ohte, Hideaki Shibata, Kazuo Isobe, Ryunosuke Tateno, Tomoki Oda, Takuo Hishi, et al. 2016. “Factors contributing to soil nitrogen mineralization and nitrification rates of forest soils in the Japanese archipelago.” *Forest Ecology and Management* 361: 382-396. https://doi.org/10.1016/j.foreco.2015.11.033

2. Hart, Stephen C., John M. Stark, Eric A. Davidson, Mary K. Firestone. 1994. “Nitrogen mineralization, immobilization, and nitrification.” In *Methods of Soil Analysis: Part 2 Microbiological and Biochemical Properties*, edited by R. W. Weaver, Scott Angle, Peter Bottomley, David Bezdicek, Scott Smith, Ali Tabatabai, Art Wollum (Soil Science Society of America, Inc.), 985-1018. https://doi.org/10.2136/sssabookser5.2.c42

3. Urakawa, Rieko, Nobuhito Ohte, Hideaki Shibata, Ryunosuke Tateno, Takuo Hishi, Keitaro Fukushima, Yoshiyuki Inagaki, et al. 2015. “Biogeochemical nitrogen properties of forest soils in the Japanese archipelago.” *Ecological Research* 30: 1-2. https://doi.org/10.1007/s11284-014-1212-8

4. Isobe, Kazuo, Yuta Ise, Hiroyu Kato, Tomoki Oda, Christian E. Vincenot, Keisuke Koba, Ryunosuke Tateno, et al. 2020. “Consequences of microbial diversity in forest nitrogen cycling: diverse ammonifiers and specialized ammonia oxidizers.” *The ISME Journal* 14: 12-25. https://doi.org/10.1038/s41396-019-0500-2

5. Isobe, Kazuo, Yuichi Suwa, Junko Ikutani, Megumi Kuroiwa, Tomoko Makita, Yu Takebayashi, Muneoki Yoh, et al. 2011. “Analytical techniques for quantifying ^15^N/^14^N of nitrate, nitrite, total dissolved nitrogen and ammonium in environmental samples using a gas chromatograph equipped with a quadrupole mass spectrometer.” *Microbes and Environments* 26: 46-53. https://doi.org/10.1264/jsme2.ME10159

6. Kuroiwa, Megumi, Keisuke Koba, Kazuo Isobe, Ryunosuke Tateno, Asami Nakanishi, Yoshiyuki Inagaki, Hiroto Toda, et al. 2011. “Gross nitrification rates in four Japanese forest soils: heterotrophic versus autotrophic and the regulation factors for the nitrification.” *Journal of Forest Research* 16: 363-373. https://doi.org/10.1007/s10310-011-0287-0

7. Caporaso, J. Gregory, Christian L. Lauber, William A. Walters, Donna Berg-Lyons, James Huntley, Noah Fierer, Sarah M. Owens, et al. 2012. “Ultra-high-throughput microbial community analysis on the Illumina HiSeq and MiSeq platforms.” *The ISME Journal* 6: 1621-1624. https://doi.org/10.1038/ismej.2012.8

8. May, Lisa A., Brenda Smiley, Michael G. Schmidt. 2001. “Comparative denaturing gradient gel electrophoresis analysis of fungal communities associated with whole plant corn silage.” *Canadian Journal of Microbiology* 47: 829-841. https://doi.org/10.1139/w01-086

9. Wei, Wei, Kazuo Isobe, Yutaka Shiratori, Tomoyasu Nishizawa, Nobuhito Ohte, Shigeto Otsuka, Keishi Senoo. 2014. “N_2_O emission from cropland field soil through fungal denitrification after surface applications of organic fertilizer.” *Soil Biology and Biochemistry* 69: 157-167. https://doi.org/10.1016/j.soilbio.2013.10.044

10. Caporaso, J. Gregory, Christian L. Lauber, William A. Walters, Donna Berg-Lyons, Catherine A. Lozupone, Peter J. Turnbaugh, Noah Fierer, et al. 2011. “Global patterns of 16S rRNA diversity at a depth of millions of sequences per sample.” *Proceedings of the National Academy of Sciences* 108: 4516-4522. https://doi.org/10.1073/pnas.1000080107

11. Tedersoo, Leho, Björn Lindahl. 2016. “Fungal identification biases in microbiome projects.” *Environmental Microbiology Reports* 8: 774-779. https://doi.org/10.1111/1758-2229.12438

12. Edgar, Robert C. 2013. “UPARSE: highly accurate OTU sequences from microbial amplicon reads.” *Nature Methods* 10: 996-998. https://doi.org/10.1038/nmeth.2604

13. Rognes, Torbjørn, Tomáš Flouri, Ben Nichols, Christopher Quince, Frédéric Mahé. 2016. “VSEARCH: a versatile open source tool for metagenomics.” *PeerJ* 4: e2584. https://doi.org/10.7717/peerj.2584

14. Estaki, Mehrbod, Lingjing Jiang, Nicholas A. Bokulich, Daniel McDonald, Antonio González, Tomasz Kosciolek, Cameron Martino, et al. 2020. “QIIME 2 enables comprehensive End-to-End analysis of diverse microbiome data and comparative studies with publicly available data.” *Current Protocols in Bioinformatics* 70: e100. <https://doi.org/10.1002/cpbi.100>

15. Quast Christian, Pruesse Elmar, Yilmaz Pelin, Gerken Jan, Schweer Timmy, Yarza Pablo, Peplies Jörg, Glöckner Frank Oliver. 2013. “The SILVA ribosomal RNA gene database project: improved data processing and web-based tools.” *Nucleic Acids Research* 41: D1. https://doi.org/10.1093/nar/gks1219.

16. Kessy Abarenkov, R Henrik Nilsson, Karl-Henrik Larsson, Andy F S Taylor, Tom W May, Tobias Guldberg Frøslev, Julia Pawlowska, et al. 2024. “The UNITE database for molecular identification and taxonomic communication of fungi and other eukaryotes: sequences, taxa and classifications reconsidered.” *Nucleic Acids Research* 52: D1. https://doi.org/10.1093/nar/gkad1039

17. McMurdie, Paul J., Susan Holmes. 2013. “phyloseq: An R package for reproducible interactive analysis and graphics of microbiome census data.” *PLoS ONE* 8: e61217. https://doi.org/10.1371/journal.pone.0061217

18. Zhang, Jiajie, Kassian Kobert, Tomáš Flouri, Alexandros Stamatakis. 2014. “PEAR: a fast and accurate Illumina Paired-End reAd mergeR.” *Bioinformatics* 30: 614-620. https://doi.org/10.1093/bioinformatics/btt593

19. Noguchi, Hideki, Takeaki Taniguchi, Takehiko Itoh. 2008. “MetaGeneAnnotator: detecting species-specific patterns of ribosomal binding site for precise gene prediction in anonymous prokaryotic and phage genomes.” *DNA Research* 15: 387-396. https://doi.org/10.1093/dnares/dsn027

20. Rice, Peter, Ian Longden, Alan Bleasby. 2000. “EMBOSS: the european molecular biology open software suite.” *Trends in Genetics* 16: 276-277. https://doi.org/10.1016/S0168-9525(00)02024-2

21. Takami, Hideto. 2024. “Functional microbial diversity: Functional genomics and metagenomics using genomaple.” In *Microbial Diversity in the Genomic Era: Functional Diversity and Community Analysis*, edited by Surajit Das, Hirak Ranjan Dash (Academic Press), 439-465. https://doi.org/10.1016/B978-0-443-13320-6.00026-3

22. Takami, Hideto, Takeaki Taniguchi, Wataru Arai, Kazuhiro Takemoto, Yuki Moriya, Susumu Goto. 2016. “An automated systemfor evaluation of the potential functionome: MAPLE version 2.1.0.” *DNA Research* 23: 467-475. <https://doi.org/10.1093/dnares/dsw030>

23. Arai, Wataru, Takeaki Taniguchi, Susumu Goto, Yuki Moriya, Hideya Uehara, Kazuhiro Takemoto, Hiroyuki Ogata, Hideto Takami. 2018. “MAPLE 2.3.0: an improved system for evaluating the functionomes of genomes and metagenomes.” *Bioscience, Biotechnology, and Biochemistry* 82: 1515-1517. <https://doi.org/10.1080/09168451.2018.1476122>

24. R Core Team. 2020. “R: A language and environmentfor statistical computing.” R Foundation for Statistical Computing, Vienna, Austria. https://www.R-project.org/.

25. Liaw, Andy, Matthew Wiener. 2001. “Classification and regression by randomForest.” *R News* 2/3: 18-22. https://journal.r-project.org/articles/RN-2002-022/

26. Ferrier, Simon, Glenn Manion, Jane Elith, Karen Richardson. 2007. “Using generalized dissimilarity modelling to analyse and predict patterns of beta diversity in regional biodiversity assessment.” *Diversity and Distributions* 13: 252-264. https://doi.org/10.1111/j.1472-4642.2007.00341.x

27. Mokany, Karel, Chris Ware, Skipton N. C. Woolley, Simon Ferrier, Matthew C Fitzpatrick. 2022. “A working guide to harnessing generalized dissimilarity modelling for biodiversity analysis and conservation assessment.” *Global Ecology and Biogeography* 31: 802-821. https://doi.org/10.1111/geb.13459

28. Bernd Bischl, Michel Lang, Jakob Bossek, Daniel Horn, Jakob Richter, Dirk Surmann. 2022. “BBmisc: miscellaneous helper functions for B. Bischl. V1.13.”

29. Tenenhaus, Michel, Vincenzo Esposito Vinzi, Yves-Marie Chatelin, Carlo Lauro. 2005. “PLS path modeling.” *Computational Statistics & Data Analysis* 48: 159-205. https://doi.org/10.1016/j.csda.2004.03.005

30. Wetzels, Martin, Gaby Odekerken-Schröder, Claudia van Oppen. 2009. “Using PLS path modeling for assessing hierarchial construct models: guidelines and impirical illustration.” *MIS Quarterly* 33: 177-195. https://doi.org/10.2307/20650284


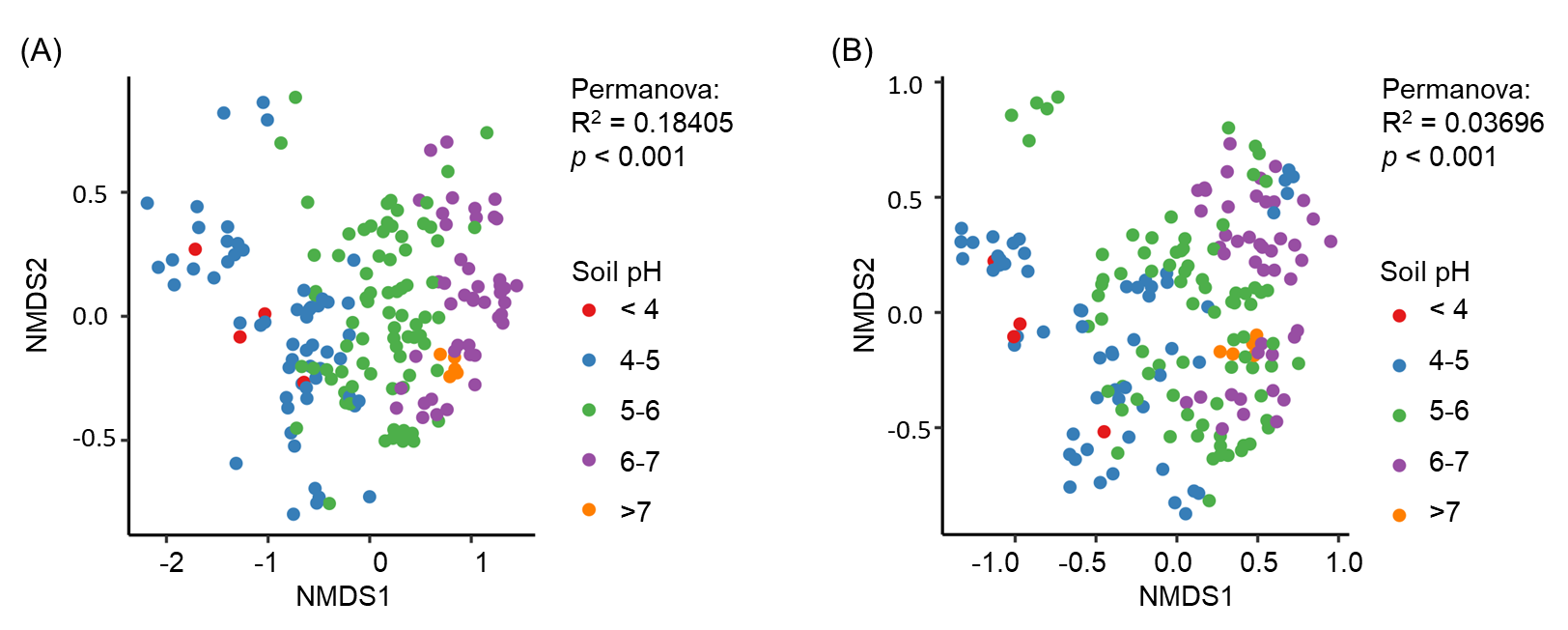


**Figure S1 Taxonomic composition shifts in response to soil habitat pH.** Taxonomic composition was determined using Bray–Curtis dissimilarity based on 16S rRNA and ITS genes for bacterial communities in panel (A) and fungal communities in panel (B). The results are plotted on non-metric multidimensional scaling (NMDS) ordination plots. Both bacterial and fungal composition correlates with soil pH.


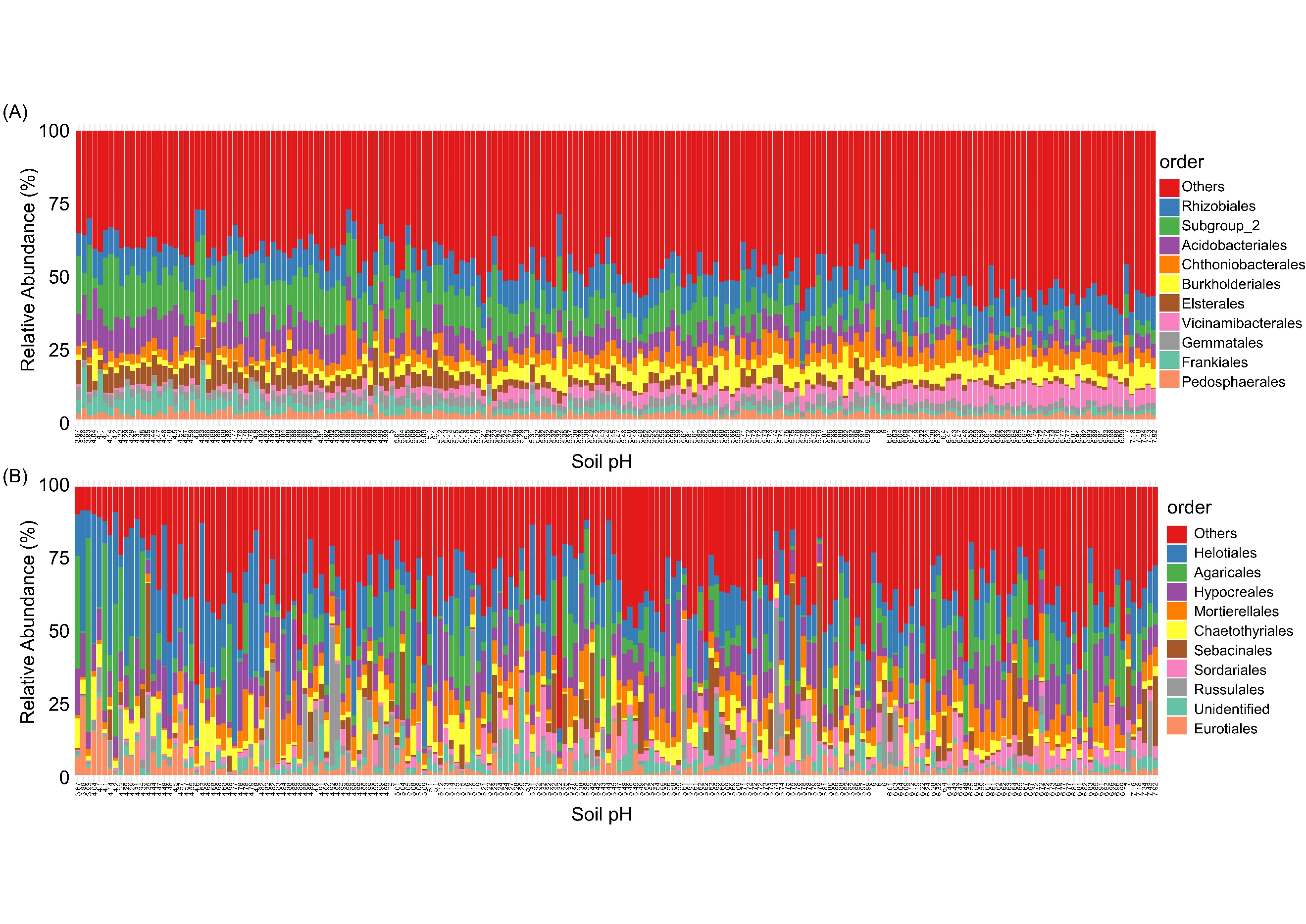


**Figure S2 Shifts in taxonomic composition of bacterial (A) and fungal (B) communities at the order level in response to soil pH.** The top 10 most abundant orders are shown, with all other taxa grouped as “Others”. A complete list of all detected orders is provided in Table S3.


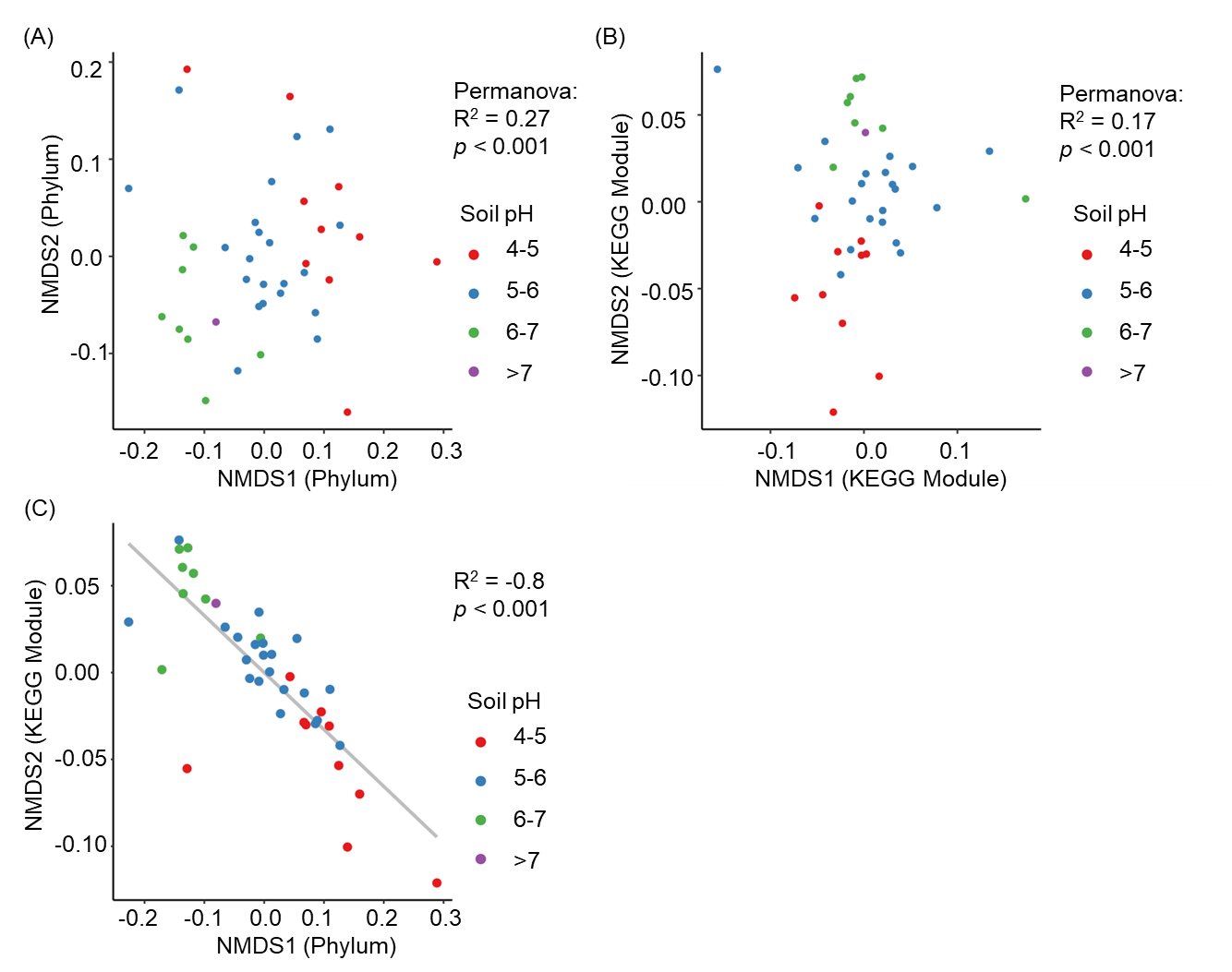


**Figure S3 Shifts in taxonomic and metabolic composition in response to soil pH.** (A and B) Taxonomic and metabolic compositions were analyzed using Bray–Curtis dissimilarity. Taxonomic composition (panel A) was identified based on ribosomal proteins, and metabolic composition (panel B) was based on the whole Kyoto Encyclopedia of Genes and Genomes (KEGG) module. Both taxonomic and metabolic compositions correlate with soil habitat pH. (C) Correlation between nonmetric multidimensional scaling 1(NMDS1) of taxonomic composition and NMDS2 of metabolic composition is shown.


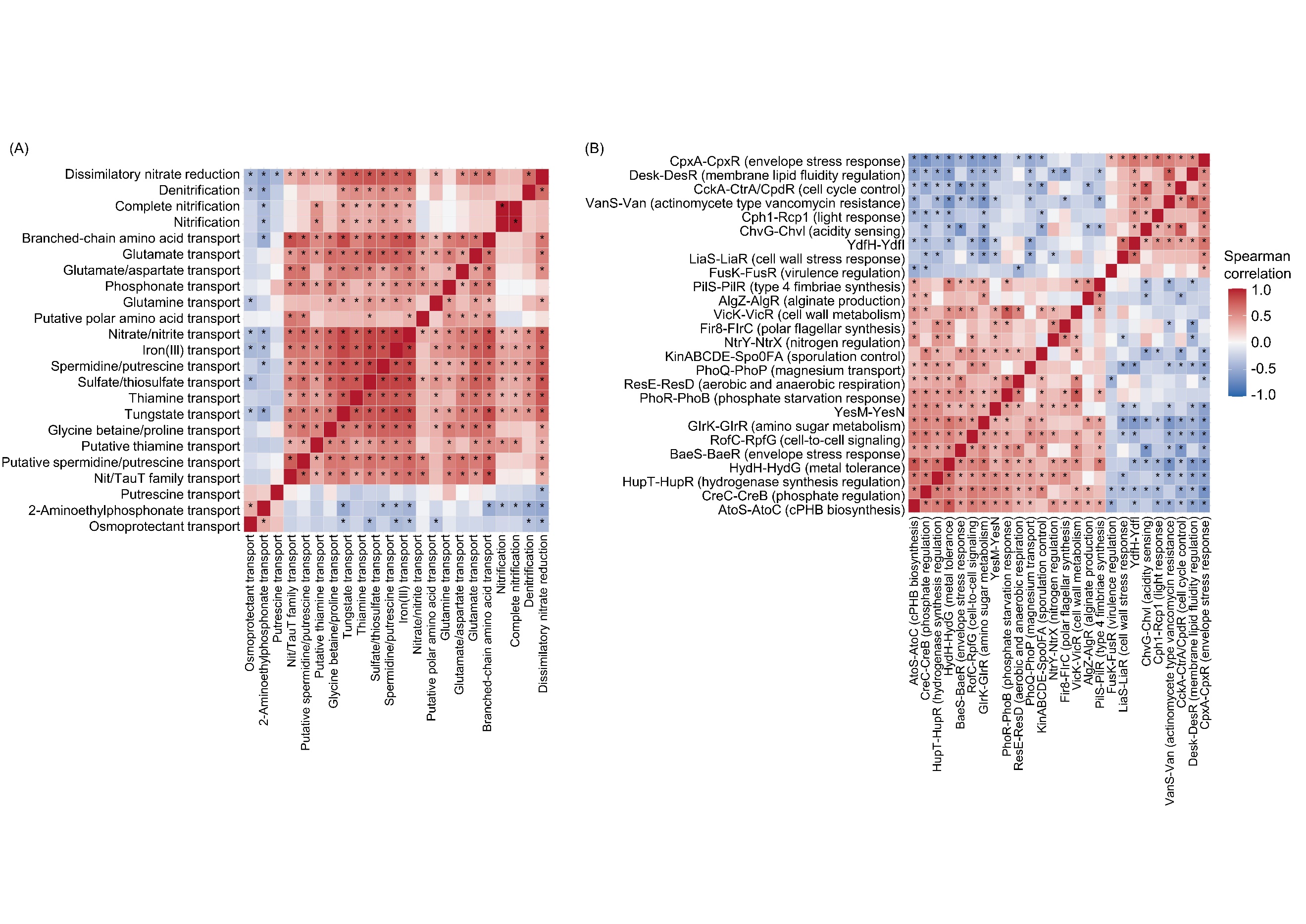


**Figure S4 Spearman correlations among KEGG modules that were significantly associated with soil pH.** (A) Kyoto Encyclopedia of Genes and Genomes (KEGG) modules involved in Mineral and organic ion transport systems, Phosphate and amino acid transport systems, and Nitrogen metabolism. (B) KEGG modules involved in the Two-component regulatory system. Asterisks indicate correlations with *p* < 0.05.
